# Functional traits help to explain half-century long shifts in pollinator distributions

**DOI:** 10.1038/srep24451

**Published:** 2016-04-15

**Authors:** Jesús Aguirre-Gutiérrez, W. Daniel Kissling, Luísa G. Carvalheiro, Michiel F. WallisDeVries, Markus Franzén, Jacobus C. Biesmeijer

**Affiliations:** 1 Naturalis Biodiversity Center, Biodiversity Dynamics, postbus 9517, 2300 RA, Leiden, The Netherlands; 2 Institute for Biodiversity and Ecosystem Dynamics (IBED), Computational Geo-Ecology, University of Amsterdam, Science Park 904, 1098 HX, Amsterdam, The Netherlands; 3 Departamento de Ecologia, Universidade de Brasília, Campus Universitário Darcy Ribeiro, Brasília, DF, 70910-900, Brazil; 4 Centre for Ecology, Evolution and Environmental Changes (CE3C), Faculdade de Ciências da Universidade de Lisboa, 1749-016 Lisboa, Portugal; 5 De Vlinderstichting/Dutch Butterfly Conservation, P.O. Box 506, 6700 AM Wageningen, The Netherlands; 6 Wageningen University, Laboratory of Entomology, P.O. Box 16, 6700AA Wageningen, The Netherlands; 7 Department of Community Ecology, UFZ, Helmholtz Centre for Environmental Research, Halle, Germany

## Abstract

Changes in climate and land use can have important impacts on biodiversity. Species respond to such environmental modifications by adapting to new conditions or by shifting their geographic distributions towards more suitable areas. The latter might be constrained by species’ functional traits that influence their ability to move, reproduce or establish. Here, we show that functional traits related to dispersal, reproduction, habitat use and diet have influenced how three pollinator groups (bees, butterflies and hoverflies) responded to changes in climate and land-use in the Netherlands since 1950. Across the three pollinator groups, we found pronounced areal range expansions (>53%) and modelled range shifts towards the north (all taxa: 17–22 km), west (bees: 14 km) and east (butterflies: 11 km). The importance of specific functional traits for explaining distributional changes varied among pollinator groups. Larval diet preferences (i.e. carnivorous vs. herbivorous/detritivorous and nitrogen values of host plants, respectively) were important for hoverflies and butterflies, adult body size for hoverflies, and flight period length for all groups. Moreover, interactions among multiple traits were important to explain species’ geographic range shifts, suggesting that taxon-specific multi-trait analyses are needed to predict how global change will affect biodiversity and ecosystem services.

Changes in climate and land use can have important effects on species distributions and may ultimately affect the supply of ecosystem services (e.g. pollination, carbon storage, clean water supplies, and pest control)^1^. Over the last decades, climate change has intensified around the world, including increasing temperatures and significant shifts in precipitation patterns and increases in the occurrence of extreme weather events^2^. Moreover, species’ habitats have changed substantially due to agricultural expansion and increased use of fertilisers, herbicides and pesticides. In some regions, large-scale land-use changes have ceased recently although highly modified landscapes with intensive agriculture and high nitrogen deposition levels remain^3^,^4^. This poses key challenges for conserving biodiversity^5^.

Shifts in species’ geographic ranges, including range contractions, range expansions and major changes in north-south and east-west distributional extents have been recently observed across the globe^6^. An in-depth understanding of these range shifts is needed for deriving essential measurements and indicators to report biodiversity change^7^,^8^, and for the development of effective conservation programmes under ongoing and future global change. While range shifts are increasingly quantified, large knowledge gaps remain in how drivers impinge on functional aspects of biodiversity and ecosystem functioning^9^. Functional traits —i.e. morphological, physiological, phenological or behavioural characteristics of species that are important for their growth, survival and reproduction— can constrain the tolerances of species and their responses to environmental changes (‘response traits’ *sensu* Díaz *et al.*^10^; see also Eskildsen *et al.*^11^). In turn, changes in species and functional trait composition can affect ecosystem functioning and service provision^9^. Hence, establishing the relationships between species distributional changes and multiple functional traits is a paramount prerequisite for predicting the consequences of global change for biodiversity and human well-being.

Insect pollinators are key to ecosystem functioning, with about 60–80% of wild plants benefiting from animal pollination^12^. Moreover, pollinators are especially sensitive to climate and land-use modifications^13^,^14^, which are key drivers causing biotic homogenisation and pollinator loss around the world^13^. In Europe, most large-scale land-use changes have taken place during the first half of the 20^th^ century (~1950), and only after 1990 more policies benefiting the environment have been implemented to counteract negative effects on biodiversity^15^. These policies have been directed to enhance (semi-) natural habitats (e.g. grasslands and forest) and —in the case of agri-environmental schemes— to increase the feeding and nesting resources for insect biodiversity in agricultural landscapes^15^. These policies can ameliorate the negative effects of climate and land-use change on insect pollinators, and may partly explain the slow-down of pollinator diversity declines in NW-Europe^16^. However, it remains unclear which species have benefitted most or least, and whether and how functional traits determine the responses of pollinators to long-term climate and land-use change.

Here, we investigate how multiple functional traits of flower-visiting insects (i.e. bees, butterflies and hoverflies; in the following referred to as ‘pollinators’) relate to changes in species distributions over a period of >50 years. We compiled a comprehensive database of pollinator occurrences from two time periods (1951–1970 vs. 1998–2014) across the Netherlands, excluding species with few sampled locations (less than 5 records) and those that have not been constantly present in the study area. This included ca. 60% of the known species of Dutch pollinators. These limits are needed for data analysis, but eliminate most of the rare, threatened species as well as those that have gone extinct or recently colonized the Netherlands. We used species distribution models (SDM) together with climate and land use data to model species’ geographic distributions at 5 × 5 km resolution. We then applied multivariate linear models with pollinator range changes between time periods as response variables (i.e. areal range changes as well as latitudinal and longitudinal shifts), and multiple functional traits as predictors including the initial range size of the species as a control variable (see methods section). The selected functional traits (Table 1) are thought to represent insects’ response traits (*sensu* Díaz *et al.*^10^) to climatic and land-use changes and are related to key aspects of their life histories (dispersal, reproduction, habitat use, and diet). Because multiple traits are often involved in species responses to both climate and land-use change (e.g. Diamond *et al.*^17^; Williams *et al.*^18^), and these can act simultaneously, we included their two-way interaction terms in all analyses.

Despite the loss of several species in the studied region during the past century^16^, given the decrease of major land-use changes in the Netherlands in recent decades^4^ and the investments into agricultural practices promoting biodiversity^15^, we expected that several pollinator species should have expanded their ranges over the studied period. In particular, we hypothesized that habitat use (i.e. degree of habitat specialisation) and diet (i.e. larval food preferences) might be key to explaining areal range changes^19^. More specifically, we predicted that habitat generalists and species with broad diets and widely distributed feeding resources should have increased their distributional extent more than species with more specialised habits. Due to increased changes in temperature and precipitation during the last half-century^20^, we further expected that observed range shifts along latitude and longitude might be related to dispersal and reproductive traits that enable species to better (and more abundantly) move across landscapes. Specifically, we expected that range shifts are more accentuated for species with large body size, long flight periods and multiple generations per year, and that species generally shift towards northern latitudes, given the recent temperature increases^2^. Interactions between traits are expected, as for example the lengths of flight period (a proxy for dispersal) may interact with traits representing habitat use so that species with long flight periods may be better able to colonise distantly located habitats. Furthermore, the length of flight period may also interact with species diet preferences because species with long flight periods can access a more varied set of feeding resources at different times of the year^21^. For longitudinal shifts, both climate and land use conditions might be relevant, and thus we expected several functional traits to be relevant for these shifts.

## Results

### Spatial changes in pollinator distributions

**Figure 1:**
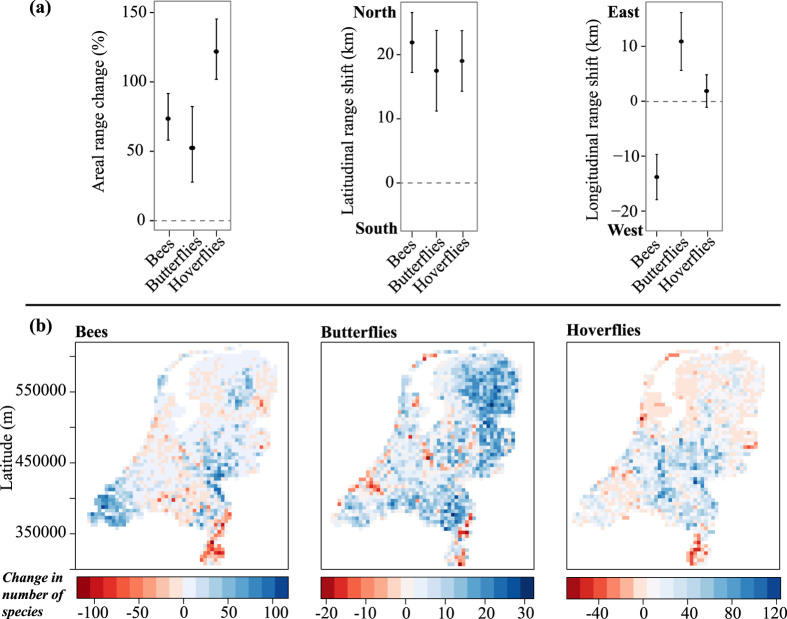
Half-century changes in species distributions of Dutch pollinators (bees, butterflies, hoverflies). (**a**) Three aspects of species distributional changes between period 1 (1951–1970) and period 2 (1998–2014) are captured. Left: areal range changes (% change in geographic range size between periods) as obtained from back-transformed values of model estimates. Middle: latitudinal range shifts (latitudinal change of the range centroid between periods, with positive values representing northward shifts and negative values southwards shifts, in km). Right: longitudinal range shifts (longitudinal change of the range centroid between periods, with positive values representing eastward shifts and negative values westward shifts, in km). For all spatial range changes, the means ± 95% confidence interval across all species within a pollinator group are presented. For statistical details see Table S1. (**b**) Maps of net changes in the number of species per grid cell and pollinator group across the Netherlands. The maps illustrate the number of species colonising a grid cell between periods minus the number of species abandoning the same grid cell. Blue colours: grid cells with more range expansions than contractions. Red colours: grid cells with more range contractions than expansions. The maps were created using the R “raster” package (https://cran.r-project.org/web/packages/raster/index.html).

From the species included in the analysis (bees: 207, butterflies: 61, hoverflies: 202), our linear models show that in the past recent decades bees expanded their modelled distributional area on average by 74%, butterflies by 53% and hoverflies by 123% (Fig. 1a left). Hence, the increases in areal ranges of hoverflies were significantly more accentuated than those of the two other pollinator groups (Fig. 1; statistical details are presented in Supplementary Table S1). From the total number of known species in the Netherlands (bees: 358, butterflies: 106, hoverflies: 328), at least 48% (bees), 44% (butterflies) and 53% (hoverflies) expanded their distributional range from period 1 (1951–1970) to period 2 (1998–2014). Nevertheless, several species presented range contractions: 33 bee species, 14 butterfly species and 25 hoverfly species (Fig. 1b, red colours; Table S5).

In addition to the areal range changes, the three pollinator groups also showed substantial latitudinal and/or longitudinal range shifts (Fig. 1a middle and right, Supplementary Table S2). Across all three pollinator groups, 79% of the analysed bee species, 77% of the butterflies and 71% of the hoverfly species have shifted northwards. Of the known Dutch species, this represents 46%, 44% and 44%, respectively. Latitudinal shifts were pronounced. On average, bees shifted 22 km northwards, butterflies 17.5 km, and hoverflies 19 km. Longitudinal range shifts (Fig. 1a right) were less pronounced than latitudinal shifts (Fig. 1a middle). Interestingly, bees and butterflies shifted in opposite directions. Most of the studied bee species (64%, representing 37% of all Dutch species) shifted significantly westwards (on average 14 km) and most butterflies (71% of analysed species, representing 41% of all Dutch species) shifted significantly eastwards (on average 11 km). Hoverflies showed a mixed picture, with 49% (32% of the Dutch species) shifting west and 51% east, resulting in an overall net change of 1.8 km.

As a result of the range change described above, increases in the number of bee species were found mostly in the south-western part, while for butterflies increasing species numbers were more frequent in the east, and for hoverflies across the central parts (Fig. 1b, blue colours). For all three taxa, species losses were detected mostly in the south-eastern region, but also in coastal areas for butterflies and hoverflies (Fig. 1b, red colours).

### Areal range changes in relation to functional traits

**Figure 2:**
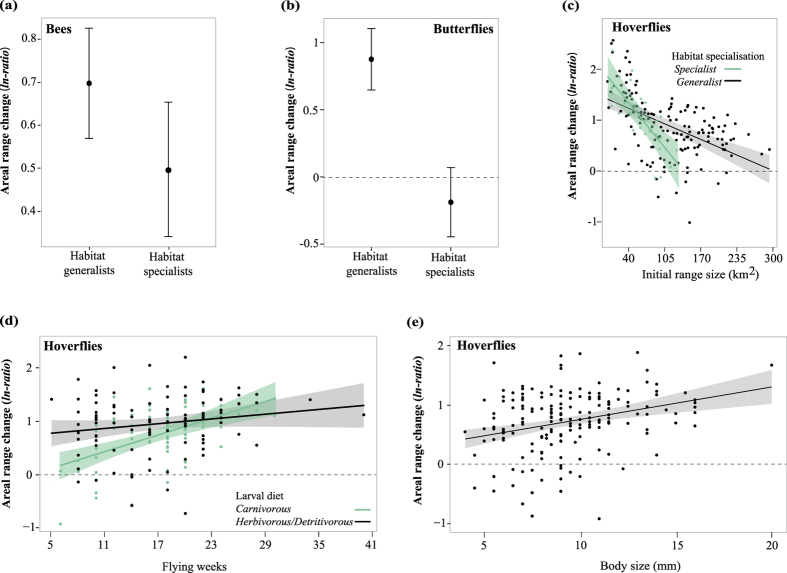
Areal range changes of pollinators (bees, butterflies, hoverflies) explained by species traits. (**a**) Bee habitat generalists show on average greater areal range expansions than specialists. (**b**) Butterfly habitat generalists show range expansions whereas habitat specialists show contractions. (**c**) Areal range expansions of hoverflies are similar in magnitude for both habitat generalists and specialists. The effect of habitat specialisation is dependent on the species initial range size. (**d**) Hoverfly’s areal range changes depend on larval food and flight period length. Areal range changes of hoverflies with larvae feeding on animals increase more strongly with flight period length than those of species with larvae feeding on other resources. (**e**) Effect of body size on areal range changes of hoverflies. Large-bodied species increase range size more strongly than small-bodied species. For all plots the average prediction ±95% confidence intervals are shown. For statistical details see Supplementary Table S3.

Several functional traits and their interactions helped to explain these range changes and distributional shifts of pollinators (Table 1). Areal range changes were mostly explained by traits related to habitat specialisation, larval feeding habits, flight period, and body size (Fig. 2; Table 2 and Supplementary Table S3). Habitat specialisation was important to explain areal range changes, but the strength of this effect differed among groups (Fig. 2a–c). As predicted, habitat generalists of bees and butterflies showed larger range expansions than habitat specialists (Fig. 2a,b). Hoverfly habitat generalists and specialists with small initial range size showed higher range increases than species with wide initial range sizes, with habitat specialists having slightly stronger increases than habitat generalists when initial range size was small (Fig. 2c). Besides habitat specialisation, larval feeding preferences also played a role for range changes, but only for hoverflies (Fig. 2d). Species with carnivorous larvae (feeding mostly on aphids) showed more pronounced areal range expansions with increasing flight period length than herbivorous/detritivorous species (Fig. 2d). Body size was only important for hoverflies for which large-bodied species showed stronger areal range expansions than small-bodied species (Fig. 2e).

### Latitudinal shifts in relation to functional traits

**Figure 3:**
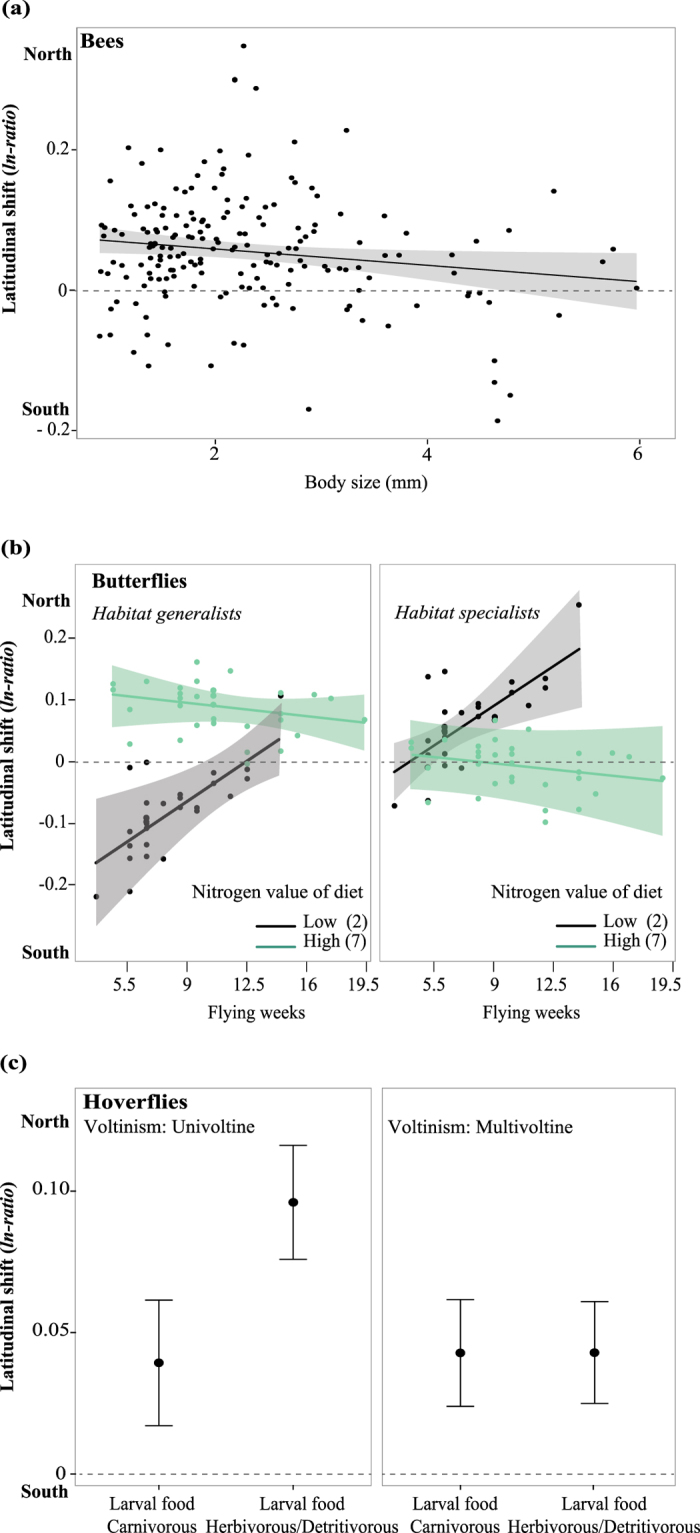
Latitudinal range shifts of pollinators (bees, butterflies, hoverflies) explained by species traits. (**a**) Effect of bee body size on latitudinal range shifts, with smaller species tending to shift more towards northern locations than larger species. (**b**) Butterfly latitudinal range shifts depend on habitat use (generalist vs. specialists), flight period length and larval host plant use. We present the model results for low (2) vs high (7) nitrophily values. Habitat generalists feeding on larval host plants with high nitrophily values (left panel: green dots) shift more towards the north than generalists feeding on plants with low nitrogen values (left panel: black dots). The opposite is observed for habitat specialists (right panel). Hence, butterfly species with larval host plants that have low nitrophily values (both panels: black dots) shift towards the south for generalists (left) but towards the north for specialists (right). Latitudinal range shifts of both specialists and generalists also depend on flight period, with northward shifts increasing with flying weeks when larval host plants have low nitrophily values, and decreasing when larval host plants have high nitrophily values. (**c**) Effect of voltinism and larval food plants on latitudinal shifts. Univoltine species (left panel) show smaller northward shifts in species which have larvae feeding on animals (carnivorous) compared to species with larval feeding on plants and organic matter (herbivorous/detritivorous. In contrast, there is no difference for multivoltine species (right panel). For all plots the average prediction ± 95% confidence intervals are shown. For statistical details see Supplementary Table S3.

Functional traits that explain latitudinal range shifts varied greatly among the three pollinator groups (Fig. 3; Table 2 and Supplementary Table S3). For bees, body size was the only trait explaining latitudinal range shifts, but the effect was weak, only explaining a small part of the variance of the data (Table 2, see 2^nd^ best model). Small-bodied bee species showed slightly stronger shifts towards northern latitudes than large-bodied species (Fig. 3a). For butterflies, both habitat specialisation and larval food preferences (i.e. nitrogen value of diet) were associated with latitudinal shifts (Table 2). Habitat generalists and species whose larvae feed on nitrophilous plants (plants adapted to high nitrogen conditions) showed stronger shifts towards northern locations than species feeding on non-nitrophilous plants (Fig. 3b, left). However, butterfly habitat specialists feeding on non-nitrophilous plants shifted their ranges more than species feeding on nitrophilous plants (Fig. 3b, right). The effect of larval feeding habits additionally interacted with flight period, with butterfly habitat generalists again showing opposite trends to habitat specialists (Fig. 3b). For hoverflies, larval food preferences (carnivorous vs. herbivorous/detritivorous) also played a role to explain latitudinal shifts, but this depended on voltinism (Fig. 3c): univoltine species (with one generation per year) with herbivorous/detritivorous larvae showed stronger shifts towards northern locations than carnivorous hoverfly species. For multivoltine hoverflies, no effect of larval diet preference could be detected.

### Longitudinal shifts in relation to functional traits

**Figure 4:**
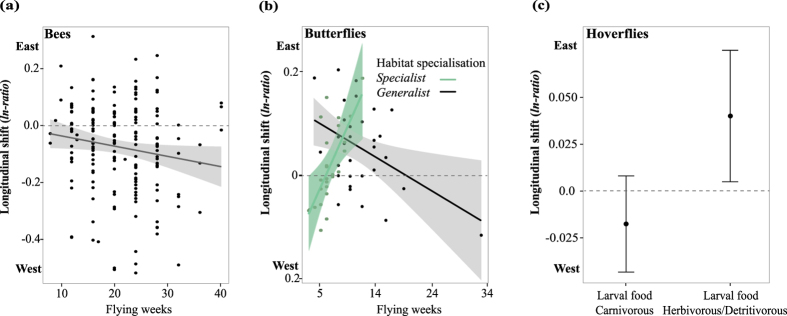
Longitudinal range shifts of pollinators (bees, butterflies, hoverflies explained by species traits. (**a**) Bee species with long flight periods show slightly stronger shifts towards western locations than species with shorter flight periods. (**b**) Longitudinal shifts of butterfly species further depend on habitat specialisation, with long-flying specialists shifting more towards eastern locations than long-flying habitat generalists. (**c**) Longitudinal shifts of hoverfly species depend on the diet of the larvae, i.e. species with herbivorous/detritivorous larvae shift towards eastern locations and species with carnivorous larvae shift towards the west. Average predictions ±95% confidence intervals are shown. For statistical details see Supplementary Table S3.

Longitudinal range shifts were also influenced by multiple functional traits, including flight period length, voltinism, habitat specialisation and larval feeding habits (Table 2 and Supplementary Table S3). For bees, species with prolonged flight periods and multiple generations per year showed stronger shifts towards western locations than univoltine species with short flight periods (Fig. 4a). However, these traits only explained a small portion of the longitudinal shifts (Table 2). For butterflies, habitat generalists that have short flight periods and habitat specialists that have longer flight periods showed the strongest shifts towards eastern locations (Fig. 4b). For hoverflies, larval feeding preference was the only trait explaining longitudinal range shifts (Fig. 4c; Table 2). Hoverflies feeding on animals (carnivorous) tended to shift towards the west whereas species feeding on other sources (herbivorous/detritivorous) tended to shift towards the east.

## Discussion

Global change profoundly modulates biodiversity, but how the intrinsic characteristics of species constrain their long-term responses to climate and land-use change remains little explored. Here, we used a comprehensive long-term dataset of species occurrences in the Netherlands to evaluate how key aspects of insect life histories affect the distributional responses of 470 pollinators (ca. 60% of the known Dutch pollinator species, but excluding species that are very rare or did not occur in the Netherlands during one of the time periods analysed) to climate and land-use change since the 1950s. Our study clearly shows that species range changes were mediated by multiple traits related to dispersal, reproduction, habitat use and diet. Moreover, different pollinator groups (bees, butterflies, hoverflies) often showed contrasting trait-mediated responses, partly driven by interactions among multiple functional traits. This suggests that taxon-specific multi-trait analyses are needed to better understand how global change affects species distributions and ecosystem functioning.

### Spatial changes in pollinator distributions

We detected range expansions for most studied species over the last half century. These range expansions may be also understood as increases in the areas with suitable environmental conditions for the pollinators here analysed but not necessarily as increases in habitat quality, area of occupancy, population size or population persistence within the modelled ranges. The detected expansions might be related to temperature increases, a slow-down of major land-use changes, and investments into practices to enhance biodiversity as agri-environmental schemes^15^,^20^,^22^,^23^,^24^. Alternatively, some of these modelled range expansions could be caused, at least partly, by an increased sampling effort over time. However, as shown in Fig. S1 (see also methods section in “Spatial changes in pollinator species distributions”), this is unlikely to have a strong effect as the sampled grid cells in period 1 and period 2 covered almost the same distributional extent. Moreover, the sampled grid cells did not highly differ in their area covered, suggesting that the observed areal range changes are unlikely to be caused by sampling bias. The recorded shifts of the majority of species (all groups) towards higher latitudes suggest that species have generally expanded northwards during the last fifty years, most likely due to global warming. Shifts detected towards the west (bees) and east (butterflies) might be more related to land use changes, but climate change could also play a role here. This requires more detailed analyses about the global change drivers underlying these range shifts. Moreover, several species from the three pollinator groups (151 of bees, 45 of butterflies and 126 of hoverflies) could not be included in this analysis due to our rigorous selection criteria (see methods). These species are likely to represent declining or rare species, or species which have only recently moved into the study area. Indeed, previous studies encompassing a larger set of species have detected local and global species richness declines in the study region during the same study period^16^. Future work should investigate in more detail the distributional trends of such species which could help to evaluate the generality of our findings.

### Areal range changes in relation to functional traits

The range expansions for bees, butterflies, and hoverflies as reported here are consistent with recently reported species richness trends which suggest a pattern of slowdown of declines and sometimes even pollinator recovery in NW-Europe^16^. As expected, habitat generalists expanded more than habitat specialists, supporting the widely observed replacement of ecological specialists by broadly adapted ecological generalists^11^. This may be driven by changes in anthropogenic habitats and decreases in (semi-)natural habitats (except forests) in the Netherlands over the studied time period^4^. The stronger expansion of habitat generalists relative to habitat specialists was observed for bees and butterflies (Fig. 2a,b). For hoverfly species the effect of habitat specialisation depended on their initial range size: the difference between habitat specialists and generalists was most pronounced for species with large initial range sizes (Table S3). In the Netherlands, the large amount of agricultural lands (which benefit aphid-feeding hoverflies) and the recovery of forest systems (which benefit saproxylic hoverflies) could explain these parallel range expansions of both hoverfly habitat specialists and generalists, and the detected effect of larval diet preferences^25^,^26^,^27^. The effects of flight period length and body size on hoverfly range expansions may reflect the lower susceptibility of species with large body size and prolonged flight periods to climate and land-use modifications (see Chown *et al.*^28^).

### Latitudinal shifts in relation to functional traits

We report pronounced half-century long range shifts towards northern latitudes for all three pollinator taxa. These findings are consistent with previously reported decadal latitudinal range shifts of other organism groups^29^. Overall, these latitudinal shifts were only moderately related to species traits of bees. Body size, the only retained predictor trait, explained only a small part of bees’ latitudinal shifts, suggesting that it may not be a strong proxy for species dispersal capacity as a response to environmental change (see Stevens *et al.*^30^). We found that larval food preferences play an important role in explaining the magnitude of latitudinal range shifts in butterflies and hoverflies, but not in bees. In contrast to bee species, butterflies and hoverflies have larvae that highly depend on resources in their immediate neighbourhood^31^,^32^. Our results for hoverflies and butterflies might therefore exemplify the importance of feeding and nesting resources for the colonisation and population persistence of these taxa, in addition to climate^33^. For butterflies with wide habitat preferences, latitudinal shifts were most accentuated if larval host plants have affinities with nitrogen-rich habitats. The Netherlands is among the countries with the highest nitrogen deposition levels worldwide^3^, which has led to strong increases of nitrophilous plants^34^ and potential consequences for development rates, reproductive potential^35^ and the distribution of many butterflies^36^. Moreover, although butterflies showed an overall expansion towards the north, the rate of expansion is slower (<50%) than the rate of climatic warming for most butterfly species^37^, which may result in a non-equilibrium of species distributions and suitable habitats. For hoverflies, the effect of larval food preferences on latitudinal shifts further depended on voltinism (i.e. number of generations per year), with univoltine herbivorous/detritivorous species showing stronger northwards shifts than other hoverflies species. This implies that plant and organic feeding resources have become more widely available in northern locations (e.g. via increases in forested ecosystem favouring saproxylic species^25^), but that species with only one, possibly long-lived, generation per year can reach these resources more easily compared to species with many, but short, generations.

### Longitudinal shifts in relation to functional traits

Range shifts along longitude have been investigated less than latitudinal range shifts^6^. We show that longitudinal shifts can be partially explained by flight period length (butterflies), habitat specialisation (butterflies), and larval diet preferences (hoverflies). For bees, the effects of traits (flight period and voltinism) on longitudinal shifts were rather weak (see Table S3), suggesting that additional traits may be important for mediating bee species responses to environmental change.

The tendency of butterflies to shift towards the east may reflect changes in (semi-) natural habitats (especially for habitat specialists), which have become less available in the west where highly populated urban centres and agricultural lands have expanded (e.g. in the Randstad area; see map in Hazeu *et al.*^23^). This might also explain why generalist butterflies occupy a wide range of conditions along the longitudinal gradient (east-west) as many of them are adapted to human-dominated areas. Moreover, the long flight periods of some butterfly species may enhance their winter survival by facilitating the access to feeding resources throughout the year^21^. In our analysis, the divergence in longitudinal trends (east or west) of hoverflies is related to their different larval diet preferences. Species with larvae feeding on animals shifted towards the west, where agriculture landscapes (and thus feeding resources for aphid feeders^26^) have become available. The shifts towards the east detected for herbivorous/detritivorous hoverfly species could be explained by an increased availability of (semi-)natural habitats such as forest ecosystems in this area^25^.

### Conclusions

Substantial changes in species distributions of the more common pollinators have occurred in the Netherlands over the last half century. This includes pronounced range expansions for many species as well as range shifts towards the north, west and east. These spatial changes in pollinator distributions have potentially affected ecosystem functions (i.e. pollination of wild plants) and ecosystem services (i.e. pollination of crops), although the specific consequences for biodiversity and human well-being remain poorly quantified. Our results show that multiple functional traits related to dispersal, reproduction, habitat use, and diet partly allow to predict such range changes, but the relevance of specific traits differs among pollinator groups. The fact that no clear relationship was found between bee species traits and their latitudinal and longitudinal range shifts suggests that other traits might be important to explain the range shifts of this group. For butterflies, and to a lesser extent for hoverflies, we show a strong relationship between the included functional traits and their spatial distributional shifts, making them good examples of trait-mediated responses to environmental changes across time. Moreover, interactions among multiple traits appear to be important to predict latitudinal and longitudinal shifts, with contrasting trends between habitat specialists and generalists or between species with different reproductive potential (e.g. univoltine and multivoltine species). We therefore suggest that more taxon-specific analyses on trait-mediated range changes are urgently needed to predict how global change will affect the future of biodiversity and human well-being. Additionally, we highlight the need for long-term monitoring programmes for pollinators and other insects, not only for common and widespread species, but also for rare and narrowly distributed species which due to their small range size and limited data availability are often not well represented in most range change analyses.

## Methods

### Study region and time periods

Our study region, the Netherlands, is located in NW-Europe, and has a temperate climate with cold winters (average minimum temperature of −1 °C) and warm summers (average maximum temperature of 24 °C)^38^. The most prominent land use systems are agriculture (55% of land area) and urban areas (www.fao.org/countryprofiles). The area has experienced major changes in climate^38^ and land use, which have been registered for more than 100 years^23^,^24^. Strong modifications of biogeochemical flows have also occurred in the last century, including major changes in phosphorus and nitrogen cycling^3^. Moreover, biodiversity in the Netherlands has been intensively studied since the early 19^th^ century.

We grouped all data (species, climate and land use) into two main time periods (TP1: 1951–1970 and TP2: 1998–2014). This was based on observed changes in climate conditions (increases in temperature and extreme weather events) and land use (high habitat fragmentation and changes to anthropogenic habitats as agriculture and urban areas around 1960)^23^,^24^. The two periods were centred in years for which land use data of high accuracy were available (TP1: 1960 and TP2: 2008; see below) and encompassed, for the species distribution data, 10 years before and after the central year in period 1 (1951–1970), and a 10 years before and 7 years after in period 2 (1998–2014). These two time periods therefore may reflect key changes in pollinator species distributions and environmental conditions across half a century.

### Species distribution data

We studied three important flower visitor groups: bees (Hymenoptera: Apoidea), butterflies (Lepidoptera: Papilionoidea and Hesperioidea), and hoverflies (Diptera: Syrphidae). The distribution data for each species were obtained for bees and hoverflies from the European Invertebrate Survey (EIS-Nederland, www.eis-nederland.nl) and for butterflies from the Dutch National Database of Flora and Fauna (NDFF, www.ndff.nl). Experts and volunteers have systematically collected the presence data over the last decades and the quality of species identification and the location accuracy of occurrence records has been assessed by the EIS and the NDFF (see www.ndff.nl/validatie). For a full description of the species collection methods see www.ndff.nl/protocollen. Since older species occurrence records have usually a higher uncertainty in their geographic location than newer records, we accounted for this uncertainty by compiling all occurrence records at a resolution of 5 × 5 km grid cells. We included only species that were present (1) in at least five 5 × 5 km grid cells, (2) in each of the two time periods, and (3) in the gap period (1971–1997). The latter was done to represent all species that have been constantly present in the study area since the 1950 s. This guarantees robust species-environment responses in the modelling process (see below) and allowed analysing the distribution patterns of a total of 207 bee species (out of 358 known species in the Netherlands), 61 butterfly species (out of a total of 106), and 202 species of hoverflies (out of a total of 328) (Supplementary Table S5). Note that given the selection criteria we had to exclude very narrowly distributed species which could potentially be threatened in the Netherlands. From the 1820 grid cells (5 × 5 km) across the Netherlands, in TP1 914 had records for bees, 894 for butterflies and 1094 for hoverflies. In TP2, the number of grid cells was 1346 for bees, 1655 for butterflies and 1592 for hoverflies. Grid cells from which samples of the three pollinator groups were extracted were distributed across all the Netherlands and across its full latitudinal and longitudinal extent (see Fig. S1).

### Species distribution modelling

For constructing species distribution models (SDMs) we extracted climate and land use data that can have an impact on the survival and distribution of pollinators. For climate, maximum, minimum and average values of temperature and precipitation per grid cell were obtained from the project “ClimateEU: historical and projected climate data for Europe”^39^. Climatic data were extracted at the same resolution as the species distribution data (5 × 5 km grid cells) and then used to calculate the 19 bioclimatic variables as described in Hijmans *et al.*^40^. After preliminary correlation analyses of all bioclimatic variables we selected four precipitation-related variables (all in mm) and five temperature related-variables (all in °C): annual precipitation, precipitation of wettest month, precipitation of driest month, precipitation of warmest quarter, mean diurnal temperature range, temperature seasonality, mean temperature of wettest quarter, mean temperature of driest quarter and mean temperature of warmest quarter. These climate variables showed low to intermediate correlation coefficients (Pearson’s correlation ≤ |0.75|) between each other.

Land use data were obtained from the geo-information department of Wageningen University (www.wageningenur.nl) for both time periods at an original resolution of 25 × 25 m. Both land use maps for period 1 and 2 had a high land use classification accuracy (~95% and 85% respectively)^23^,^24^. The land use maps were obtained for the years 1960 (representing period 1) and 2008 (period 2), which both are the central points in each time period for which the species distribution data were aggregated. The newer land use map had a higher thematic resolution than the older land use map. Hence, both maps were reclassified to make land use types consistent between time periods. Eight land use types were extracted: agriculture, grassland, forest, moors/peat, sandy soils, swamps, urban and water. Based on these reclassified land use maps we calculated for each 5 × 5 km grid cell and time period a total of twelve land use metrics that have been shown to impact the distribution and richness of pollinators^41^: percentage of each land use class (for the eight classes), number of land use classes, total edge density (m/ha), average patch area of suitable habitat (ha) and the edge density between managed and natural systems (m/ha). These metrics characterized three major aspects of landscape and habitat structure^42^: landscape composition (nine metrics), habitat fragmentation (two metrics) and spillover potential (one metric) (see below).

For landscape composition, the calculated metrics reflected the percentage of the eight land use types per grid cell as well as the total number of land use classes per grid cell (one metric). The total number of land use classes was included as a proxy of spatial heterogeneity, which can influence the turnover of pollinator species assemblages^42^. We represented habitat fragmentation with two metrics: the average area of suitable habitat patches and total edge density (total length of edges per hectare). For the former, we classified the land use classes grassland, moors/peat, forest and sandy soils as suitable habitat, and agriculture, urban, water and swamps as non-suitable habitat^43^. For the latter, we calculated the density of edges between all land use types in a grid cell. We used an additional metric to characterize species spillover potential, i.e. the potential for movements of organisms across managed and natural systems^44^. For this, we considered the land use types grassland and agriculture as (intensively-)managed, and the land use types moors/peat, forest, swamps and sandy soils as (semi-)natural systems. We then calculated the edge density between these two systems. Urban and water were not taken into account in this calculation. All calculations of land use metrics were carried out in R (Development Core Team, http://cran.r-project.org) with the “SDMTools” package.

With the data on species occurrences, climate and land use we constructed SDMs for each of the 407 bee, butterfly and hoverfly species for both period 1 and 2 using MaxEnt^45^. MaxEnt is a machine learning modelling technique with high model accuracy that has been extensively used for modelling large sets of species in locations with contrasting environmental conditions^46^. We selected MaxEnt after comparing it with other SDM algorithms (generalized boosting models, generalized linear models, random forest, artificial neural networks) for modelling a range of species with different sample sizes and different geographic distributions within the same study area, as it was one of the best performing algorithms with high model sensitivity and specificity^47^. In MaxEnt, we allowed the use of different feature types depending on the number of records available as described in Elith *et al.*^48^. As species sampling collections are often geographically biased, this can also create bias in the environmental gradient selection. To account for this, we followed Phillips *et al.*^49^ and only extracted background information for SDMs from those collection localities where species from the same pollinator group had been sampled. This procedure has been shown to greatly increase model performance (“target group approach”)^49^. It further aids to account for possible sampling and environmental selection biases because the modelled data contains the same collection bias as the data used for the background selection^48^. We computed SDMs for each species using ten repetitions with a bootstrap approach where 80% of the data was used for training and 20% for model testing. In order to account for within algorithm model variation, we obtained an ensemble model for each species by averaging the suitability scores across the ten model repetitions and used this ensemble model in subsequent analysis. Model performance per species was summarized with the area under the curve (AUC) values of the receiver-operating characteristic^50^. AUC is a common measure of SDM performance with values ranging from 0 to 1 (higher scores represent higher model accuracy). All implemented SDMs showed high accuracy (average AUC ± SD: 0.81 ± 0.09) across species and periods (Supplementary Table S5). We applied the MaxEnt logistic output format to convert the ensemble suitability maps into binary maps (presence-absence) using the threshold that maximises the sensitivity and specificity of the model^51^. These binary distribution maps were then used to analyse the spatial changes in pollinator distributions (see below).

### Spatial changes in pollinator species distributions

We quantified three different aspects of spatial range changes based on modelled species distributions between the two time periods: (1) areal range changes (contractions and expansions), (2) latitudinal range shifts, and (3) longitudinal range shifts. Areal range changes were calculated between time periods as the percentage gain or the percentage loss in geographic range size of each species using the “biomod2” R package (http://cran.r-project.org). We used linear models with Gaussian error structure to test if areal range changes of pollinator groups differed significantly from zero and between time periods using the pollinator group (bees, butterflies and hoverflies) as explanatory variable. To normalize residuals, we used the natural log of the ratio of areal range change as response variable. We then used a post-hoc pairwise comparison test (TukeyHSD) to assess whether the three pollinator groups differed significantly in areal range changes between the two time periods.

To assess latitudinal and longitudinal range shifts (north-south, east-west), we used the centroids of the predicted (binary) species distribution maps for period 1 and 2 and calculated the difference in latitudinal and longitudinal location (in kilometres). This was done using the directional distribution tool in ArcGIS (v10.1 ESRI Redlands, CA). Values of zero reflect no change in the latitudinal or longitudinal midpoint of a species geographic range between periods, values above zero indicate range shifts towards northern or eastern locations, and values below zero represent range shifts towards southern or western locations. We applied Students *t*-tests for each pollinator group to quantify whether differences in latitudinal or longitudinal midpoints differed significantly between the two time periods.

The sampling intensity could potentially affect the results obtained for areal range changes and geographic distributional shifts. For instance, a different representation of available environmental conditions between time periods might affect the outcomes of species distribution models. We therefore investigated if the sampled grid cells in TP1 and TP2 were distributed across the same latitudinal and longitudinal extent. The result showed a similar and consistent latitudinal and longitudinal distribution of the sampled grid cells across time periods (see Fig. S1). We further quantified the area of the ellipsoid containing 95% of the sampled grid cells in TP1 and in TP2 to test if the reported areal range changes may be affected by a wider distribution of grid cells in TP2 compared to TP1. Differences in the area of the ellipsoids of sampled grid cells were similar for bees (TP1 = 73513.5 km^2^; TP2 = 76556.5 km^2^), butterflies (TP1 = 69872.3 km^2^; TP2 = 74416.1 km^2^), and hoverflies (TP1 = 74034.4 km^2^; TP2 = 74132.2 km^2^). These additional analyses suggest that sampling bias is unlikely to be of major importance for the reported areal range changes and geographic range shifts.

### Statistical analysis of distributional changes in relation to functional traits

We applied multivariate linear models with a Gaussian error structure to analyse if and to what extent species’ functional traits (see Table 1) can explain half-century distributional changes of Dutch pollinators. To investigate the possible multicollinearity problems between all functional traits we calculated the generalized variance inflation factor (GVIF)^52^. This showed that all variables had GVIF values < 3.2. Hence, the GVIF values were below a commonly used threshold of 10^53^, indicating acceptable levels of collinearity for linear model analysis (Table S6). To normalize residuals, we used the natural log of the ratio of areal range change, and the latitudinal as well as the longitudinal centroid shifts between time periods as response variables. We tested for all two-way interactions between predictor variables because combinations of functional traits may be involved in species responses to climate and land-use modifications. Because initial range size (number of occupied 5 × 5 km cells in period 1) might constrain distributional responses to global change, especially when analysing relative values (i.e. species that have very small initial ranges are more likely to double their range than species occupying a greater area), we included it as a control explanatory variable. Initial range sizes of species as predicted by SDMs covered less than 27% of the land area of the Netherlands (median: 7%; range: 0.14–27%). All continuous explanatory variables were standardized and centred before analysis. We selected the most parsimonious model based on the Bayesian Information Criteria (BIC) using the R package “MuMIn”. Stepwise backward and forward model selection based on the BIC criteria was chosen because the number of degrees of freedom was very high and, in comparison to AIC, this method penalizes more complex models by excluding terms that only explain low amounts of data variability^54^. For comparison, we also kept all candidate models with Δ BIC lower than 2. Where relevant, we also show the results of the second best model (see results section).

## Additional Information

**How to cite this article**: Aguirre-Gutiérrez, J. *et al.* Functional traits help to explain half-century long shifts in pollinator distributions. *Sci. Rep.* **6**, 24451; doi: 10.1038/srep24451 (2016).

## Supplementary Material

Supplementary Information

## Figures and Tables

**Table 1:** Characteristics of functional traits of pollinators (bees, butterflies, hoverflies) and their relation to global change drivers (climate and land use change).

Trait	Trait category	Type	Global change driver	Units	Description	Reference
Body size	Dispersal	Continuous	Climate/Land use	Millimetres	Intertegular span (bees), wing span (butterflies), body length (hoverflies)	55, 56, 57, 58
Flight period	Reproduction/Dispersal	Continuous	Climate/Land use	Count	Number of weeks flying per year	55, 56, 57,59
Voltinism	Reproduction	Categorical	Climate	Univoltine or multivoltine	Number of generations per year (i.e. number of completed life cycles): univoltine (one generation) vs. multivoltine (≥2 generations)	55, 56, 57,59
Habitat specialisation	Habitat use	Categorical	Land use	Specialist or generalist	Whether habitat use is restricted or not: one habitat type vs. several (bees); predominant association with anthropogenic CORINE habitat types (agricultural and urban as: generalists) or not (semi-natural habitats: specialists) (butterflies); number of CORINE macro habitats to which adults are mainly associated: one vs several (hoverflies).	55,57,59,60
Larval food preference	Diet	Categorical/Continuous	Land use	Polyphagous vs. non-polyphagous (bees); rank values 1−4 (butterflies); carnivorous vs. herbivorous/detritivorous (hoverflies)	Diet preference of larvae: bee’s lectic preference (related to pollen and nectar resources), with non-polyphagous representing mono- and oligo-lectic species and polyphagous representing poly-lectic species. For butterflies, number of host plants (1: monophagous; 2 oligophagous; 3: polyphagous -multiple species, 1 plant family; 4: polyphagous -multiple species,>1 plant family). For hoverflies, whether feeding on living animals: carnivorous or other: herbivorous and detritivorous.	55,57,59
Larval food dependence on nitrogen	Diet	Continuous	Land use	Ellenberg nitrogen value (only available for butterflies)	Nitrogen value of host plants: average Ellenberg nitrogen indicator values of butterflies’ larval host plants (describing soil fertility conditions and nitrogen preferences).	61, 62, 63, 64, 65

Traits are grouped into four trait categories (dispersal, reproduction, habitat use, and diet). The presented traits are hypothesized to be “response” traits (*sensu* Díaz *et al.*^10^) to one or the two global change drivers presented.

**Table 2:** Effects of pollinator functional traits on areal range changes and shifts along latitude and longitude.

	Pollinator group	Best models	Explanatory variables selected				Adj. R^2^	BIC	Δ BIC
Areal range change	*Bees*	*1*	H	IR	−	−	0.23	273.1	
		*2*	−	IR	−	−	0.21	274.2	1.1
	*Butterflies*	*1*	H	−	−	−	0.39	132.4	
	*Hoverflies*	*1*	H × IR	F × LDP	S	−	0.37	305.1	
		*2*	H	F × LDP	S	IR	0.34	305.3	0.2
Latitudinal shift	*Bees*	*1*	IR	−	−	−	0.05	−404.2	
		*2*	S	IR	−	−	0.07	−403.7	0.5
	*Butterflies*	*1*	H × ND	H × IR	F × ND	−	0.37	−175.5	
		*2*	H × LDP	H × IR	−	−	0.31	−175.3	0.2
	*Hoverflies*	*1*	V × LDP	IR	−	−	0.21	−452.6	
Longitudinal shifts	*Bees*	*1*	−	−	−	−	−	−143.4	
		*2*	F	−	−	−	0.02	−142.5	1
		*3*	V	−	−	−	0.02	−142.1	1.3
	*Butterflies*	*1*	H × F	F × IR			0.31	−119.9	
	*Hoverflies*	*1*	LDP	IR	−	−	0.08	−240	

The most parsimonious model selected by means of the Bayesian Information Criteria (BIC) is shown together with other models with a ΔBIC < 2 for each group of pollinators (bees, butterflies, hoverflies). For a detailed version of the table see Supplementary Table S3.

*F: Flight period; LDP: Larval diet preference; H: Habitat specialisation; ND: Larval diet preference related to Ellenberg nitrogen value of food.*

*plant; S: Body size; V: Voltinism; IR: Initial range size.*
