# Supplementary material for: Functional traits help to explain half-century long shifts in pollinator distributions
Source: Sci Rep. 2016 Apr 15;6:24451. doi: 10.1038/srep24451 (PMC4832205; doi:10.1038/srep24451)
Supplement: Supplementary Information [file srep24451-s1.doc]

**SUPPLEMENTARY INFORMATION**

**Functional traits help to explain half-century long shifts in pollinator distributions**

Jes­ús Aguirre-Gutiérrez, W. Daniel Kissling, Luísa G. Carvalheiro, Michiel F. WallisDeVries, Markus Franzén, Jacobus C. Biesmeijer

| **Table S1. Comparisons of areal range changes of pollinators across time.** In "Comparisons", TP refers to the time periods between which areal range changes are compared (TP 1 = 1951–1970; TP 2 = 1998–2014). "Group" represents the pollinator group, bees (B), butterflies (BU) and hoverflies (H). The first three rows test if there was a significant change between the areal range size of TP1 and TP2 for each pollinator group. The last three rows test whether the areal range changes significantly differ among pollinator groups. The coefficients from the linear models are presented. | | | | | |
| --- | --- | --- | --- | --- | --- |
| Comparisons | Group | Coefficient | Std. Error | z-value | P-value |
| TP 1 to 2 | B | 0.55 | 0.05 | 11.28 | <0.001 |
| TP 1 to 2 | BU | 0.42 | 0.09 | 4.67 | <0.001 |
| TP 1 to 2 | H | 0.8 | 0.05 | 16.09 | <0.001 |
| Range Δ TP 1 vs 2 | B vs BU | 0.13 | 0.1 | 1.28 | 0.54 |
| Range Δ TP 1 vs 2 | B vs H | -0.25 | 0.07 | -3.52 | 0.002 |
| Range Δ TP 1 vs 2 | BU vs H | -0.38 | 0.1 | -3.65 | 0.001 |
| Adjusted Tukey's *P-values* reported; Δ: Change. | | | | | |

| **Table S2**. **Results of the comparison of shifts in the midpoints of species distributions per pollinator group for the whole study period, TP1–TP2 (TP 1 = 1951–1970; TP 2 = 1998–2014).** A Student *t*-test was used to investigate if the overall changes in the centroids of the species geographic ranges differed significantly from zero (no change) within each pollinator group. | | | | |
| --- | --- | --- | --- | --- |
| Group | Latitude | P-value | Longitude | P-value |
|  | *t* |  | *t* |  |
| Bees | 4.99 | <0.001 | -4.99 | <0.001 |
| Butterflies | 2.01 | 0.04 | 2.21 | 0.03 |
| Hoverflies | 4.46 | <0.001 | 0.79 | 0.43 |

| **Table S3. Detailed results of best models to explain the relation between species functional traits of three pollinator groups (bees, butterflies, hoverflies) and areal range changes, latitudinal and longitudinal shifts, respectively.** The starting model contained areal range change, latitudinal or longitudinal shift values as response variable and single terms and all two-way interactions between functional traits as predictors (including also the starting range size). After model selection, only models with BIC Δ <2 were kept. The three best models are shown. The level of the factorial variable to which the coefficient refers to is shown in parenthesis. Coefficients and (**-/+**) 95% confidence intervals are provided for each model. | | | | | | | | | | | | | | |  |
| --- | --- | --- | --- | --- | --- | --- | --- | --- | --- | --- | --- | --- | --- | --- | --- |
|  |  |  | **Model 1** | | | | **Model 2** | | | | **Model 3** | | | | |
| **Areal range change** |  |  | **Estimate** | **95% CI** | | | **Estimate** | **95% CI** | | | **Estimate** | **95% CI** | | | |
|  | **Bees** | Intercept | 0.69 | 0.57 | ; | 0.81 | 0.56 | 0.50 | ; | 0.62 |  |  |  |  | |
|  |  | H (specialists) | -0.19 | -0.33 | ; | -0.05 |  |  |  |  |  |  |  |  | |
|  |  | IR | -0.27 | -0.33 | ; | -0.21 | -0.25 | -0.31 | ; | -0.19 |  |  |  |  | |
|  |  | *Adj. R^2^* | 0.23 |  |  |  | 0.21 |  |  |  |  |  |  |  | |
|  |  |  |  |  |  |  |  |  |  |  |  |  |  |  | |
|  | **Butterflies** | Intercept | 0.88 | 0.66 | ; | 1.10 |  |  |  |  |  |  |  |  | |
|  |  | H (specialists) | -1.06 | -1.39 | ; | -0.73 |  |  |  |  |  |  |  |  | |
|  |  | *Adj. R^2^* | 0.39 |  |  |  |  |  |  |  |  |  |  |  | |
|  |  |  |  |  |  |  |  |  |  |  |  |  |  |  | |
|  | **Hoverflies** | Intercept | 0.72 | 0.60 | ; | 0.84 | 0.71 | 0.61 | ; | 0.81 |  |  |  |  | |
|  |  | S | 0.15 | 0.09 | ; | 0.21 | 0.17 | 0.11 | ; | 0.23 |  |  |  |  | |
|  |  | F | 0.31 | 0.19 | ; | 0.43 | 0.32 | 0.20 | ; | 0.44 |  |  |  |  | |
|  |  | LDP (herbivorous and detritivorous) | 0.21 | 0.07 | ; | 0.35 | 0.23 | 0.09 | ; | 0.37 |  |  |  |  | |
|  |  | IR | -0.3 | -0.38 | ; | -0.22 | -0.33 | -0.41 | ; | -0.25 |  |  |  |  | |
|  |  | H (specialists) | -0.48 | -0.79 | ; | -0.17 | -0.12 | -0.34 | ; | 0.10 |  |  |  |  | |
|  |  | F × LDP (herbivorous and detritivorous) | -0.22 | -0.36 | ; | -0.08 | -0.25 | -0.39 | ; | -0.11 |  |  |  |  | |
|  |  | H (specialists) × IR | -0.58 | -0.95 | ; | -0.21 |  |  |  |  |  |  |  |  | |
|  |  | *Adj. R^2^* | 0.37 |  |  |  | 0.34 |  |  |  |  |  |  |  | |
|  |  |  |  |  |  |  |  |  |  |  |  |  |  |  | |
| **Latitudinal shifts** |  |  |  |  |  |  |  |  |  |  |  |  |  |  | |
|  | **Bees** | Intercept | 0.05 | 0.04 | ; | 0.06 | 0.05 | 0.04 | ; | 0.06 |  |  |  |  | |
|  |  | IR | -0.01 | -0.02 | ; | 0.00 | -0.01 | -0.02 | ; | -0.0002 |  |  |  |  | |
|  |  | S |  |  |  |  | -0.01 | -0.02 | ; | -0.0002 |  |  |  |  | |
|  |  | *Adj. R^2^* | 0.05 |  |  |  | 0.07 |  |  |  |  |  |  |  | |
|  |  |  |  |  |  |  |  |  |  |  |  |  |  |  | |
|  |  |  |  |  |  |  |  |  |  |  |  |  |  |  | |
|  | **Butterflies** | Intercept | 0.03 | 0.01 | ; | 0.05 | 0.04 | 0.02 | ; | 0.06 |  |  |  |  | |
|  |  | F | 0.03 | 0.01 | ; | 0.05 |  |  |  |  |  |  |  |  | |
|  |  | ND | 0.05 | 0.03 | ; | 0.07 | 0.03 | 0.01 | ; | 0.05 |  |  |  |  | |
|  |  | H (specialists) | 0.01 | -0.03 | ; | 0.05 | -0.01 | -0.05 | ; | 0.03 |  |  |  |  | |
|  |  | IR | -0.005 | -0.02 | ; | 0.01 | -0.005 | -0.02 | ; | 0.01 |  |  |  |  | |
|  |  | F × ND | -0.03 | -0.05 | ; | -0.01 |  |  |  |  |  |  |  |  | |
|  |  | H (specialists) × ND | -0.08 | -0.12 | ; | -0.04 | -0.05 | -0.09 | ; | -0.01 |  |  |  |  | |
|  |  | H (specialists) × IR | 0.04 | 0.02 | ; | 0.06 | 0.04 | 0.02 | ; | 0.06 |  |  |  |  | |
|  |  | *Adj. R^2^* | 0.37 |  |  |  | 0.31 |  |  |  |  |  |  |  | |
|  |  |  |  |  |  |  |  |  |  |  |  |  |  |  | |
|  | **Hoverflies** | Intercept | 0.04 | 0.02 | ; | 0.06 |  |  |  |  |  |  |  |  | |
|  |  | LDP (herbivorous and detritivorous) | 0.0001 | -0.02 | ; | 0.02 |  |  |  |  |  |  |  |  | |
|  |  | V (univoltine) | -0.003 | -0.02 | ; | 0.02 |  |  |  |  |  |  |  |  | |
|  |  | IR | -0.02 | -0.03 | ; | -0.01 |  |  |  |  |  |  |  |  | |
|  |  | LDP (herbivorous and detritivorous.) × V (univoltine) | 0.05 | 0.01 | ; | 0.09 |  |  |  |  |  |  |  |  | |
|  |  | *Adj. R^2^* | 0.21 |  |  |  |  |  |  |  |  |  |  |  | |
|  |  |  |  |  |  |  |  |  |  |  |  |  |  |  | |
| **Longitudinal shifts** |  |  |  |  |  |  |  |  |  |  |  |  |  |  | |
|  | **Bees** | Intercept | -0.07 | -0.09 | ; | -0.05 | -0.07 | -0.09 | ; | -0.05 | -0.13 | -0.19 | ; | -0.07 | |
|  |  | F |  |  |  |  | -0.02 | -0.04 | ; | -0.0004 |  |  |  |  | |
|  |  | V (univoltine) |  |  |  |  |  |  |  |  | 0.06 | 0.001 | ; | 0.12 | |
|  |  | *Adj. R^2^* | 0 |  |  |  | 0.02 |  |  |  | 0.02 |  |  |  | |
|  |  |  |  |  |  |  |  |  |  |  |  |  |  |  | |
|  | **Butterflies** | Intercept | 0.05 | 0.03 | ; | 0.07 |  |  |  |  |  |  |  |  | |
|  |  | F | -0.04 | -0.06 | ; | -0.02 |  |  |  |  |  |  |  |  | |
|  |  | H (specialists) | 0.03 | -0.03 | ; | 0.09 |  |  |  |  |  |  |  |  | |
|  |  | IR | 0.04 | 0.02 | ; | 0.06 |  |  |  |  |  |  |  |  | |
|  |  | H (specialists) × F | 0.16 | 0.08 | ; | 0.24 |  |  |  |  |  |  |  |  | |
|  |  | F × IR | 0.07 | 0.03 | ; | 0.11 |  |  |  |  |  |  |  |  | |
|  |  | *Adj. R^2^* | 0.31 |  |  |  |  |  |  |  |  |  |  |  | |
|  |  |  |  |  |  |  |  |  |  |  |  |  |  |  | |
|  | **Hoverflies** | Intercept | -0.01 | -0.03 | ; | 0.01 |  |  |  |  |  |  |  |  | |
|  |  | LDP (herbivorous and detritivorous) | 0.06 | 0.02 | ; | 0.10 |  |  |  |  |  |  |  |  | |
|  |  | IR | 0.03 | 0.01 | ; | 0.05 |  |  |  |  |  |  |  |  | |
|  |  | *Adj. R^2^* | 0.08 |  |  |  |  |  |  |  |  |  |  |  | |
| *F: Flight period; LDP: Larval diet preference; H: Habitat; ND: Larval diet preference related to Ellenberg nitrogen value of food plants; S: Size; V: Voltinism; IR: Initial range.* | | | | | | | | | | | | | | |  |
| M1–M4: Coefficients of the best models obtained; SE: standard error. | | | | | | | | | | | | | | |  |

| **Table S4. The environmental variables included in the analyses and their changes over time in the Netherlands.** Comparisons were performed with a Student *t*-tests for environmental changes between time period 1 (1951–1970) and time period 2 (1998–2014). | | | | | | |
| --- | --- | --- | --- | --- | --- | --- |
| Environmental variable | Average value | | Standard deviation | | TP1 vs TP2 | *P-value* |
|  | TP1 | TP2 | TP1 | TP2 | *t* |  |
| Mean diurnal range | 7.05 | 7.25 | 0.67 | 0.75 | 8.02 | <0.001 |
| Temperature seasonality | 5.79 | 5.66 | 0.13 | 0.14 | -27.66 | <0.001 |
| Mean temp. of wettest quarter | 15.45 | 15.85 | 0.68 | 2.06 | 7.54 | <0.001 |
| Mean temp. of driest quarter | 5.79 | 9.6 | 1.78 | 1.97 | 58.76 | <0.001 |
| Mean temp. of warmest quarter | 15.92 | 17.16 | 0.36 | 0.45 | 88.32 | <0.001 |
| Annual precipitation | 803.9 | 828.2 | 25.14 | 21.82 | 29.84 | <0.001 |
| Precipitation of wettest month | 102.28 | 91.18 | 5.96 | 4.86 | -59.09 | <0.001 |
| Precipitation of driest month | 44.84 | 41.13 | 2.91 | 3.45 | -33.65 | <0.001 |
| Precipitation of warmest quarter | 253.4 | 238.78 | 11.7 | 11.48 | -36.46 | <0.001 |
| Average patch area of suitable habitat | 104.97 | 16.61 | 354.79 | 113.3 | -9.43 | <0.001 |
| Total edge density | 0.019 | 0.023 | 0.008 | 0.011 | 11.73 | <0.001 |
| Edge density managed-natural systems | 0.007 | 0.011 | 0.004 | 0.005 | 18.37 | <0.001 |
| Number of land use classes | 6.28 | 7.72 | 1.21 | 0.75 | 40.36 | <0.001 |
| Grassland | 42.23 | 40 | 26.26 | 20.35 | -2.67 | <0.01 |
| Agriculture | 31.41 | 27.46 | 25.01 | 22.31 | -4.68 | <0.001 |
| Moors/peat | 2.04 | 1.34 | 6.32 | 4.68 | -3.53 | <0.001 |
| Forest | 8.53 | 11.14 | 13.92 | 14.23 | 5.2 | <0.001 |
| Urban | 6.94 | 10.9 | 7.69 | 10.13 | 12.39 | <0.001 |
| Water | 6.97 | 6.42 | 15.46 | 10.7 | -1.16 | 0.25 |
| Swamps | 0.2 | 1.43 | 0.7 | 5.8 | 8.37 | <0.001 |
| Sandy soils | 1.68 | 1.31 | 9.1 | 7.23 | -1.26 | 0.21 |

| **Table S5. List of included species from three pollinator groups.** The Red List status is given to show the threat level of the included species. For bees, the conservation status was obtained from Peeters and Reemer ^1^ (pages 17-21). For butterflies, the red list status was obtained from the Dutch red list of butterflies (www.vlindernet.nl/doc/De_nieuwe_Rode_Lijst_Dagvlinders.pdf). For hoverflies, the red list status was obtained from Reemer *et al.* ^2^ (page 86). The number of records available in each time periods (TP1, TP2) used during the MaxEnt modelling is provided. The area under the curve value (AUC) value represents the model accuracy (see methods) for each species and time period. The number of presence records represents the number of 5 x 5 km grid cells for which the species was reported at least once during the period analysed. | | | | | | | | | |
| --- | --- | --- | --- | --- | --- | --- | --- | --- | --- |
| **Group** | **Species name** | **Red List status Netherlands*** | **Number of presence records** | | **MaxEnt model AUC** | | **Areal range changes (%)** | **Latitudinal change (km)** | **Longitudinal change (km)** |
|  |  |  |  |  |  |  |  |  |  |
|  |  |  | **TP1** | **TP2** | **TP1** | **TP2** | **TP1-TP2** |  |  |
| Bees | *Andrena angustior* | NL | 53 | 72 | 0.86 | 0.8 | 168.29 | 73.99 | 12.9 |
| Bees | *Andrena apicata* | VU | 30 | 29 | 0.78 | 0.9 | -2.63 | 15.54 | -20.17 |
| Bees | *Andrena argentata* | T | 28 | 19 | 0.88 | 0.85 | -47.69 | 41.27 | -21.22 |
| Bees | *Andrena barbilabris* | NL | 136 | 249 | 0.76 | 0.72 | 114.55 | 1.3 | -14.5 |
| Bees | *Andrena bicolor* | NL | 30 | 169 | 0.84 | 0.8 | 340 | 63.41 | -62.62 |
| Bees | *Andrena bimaculata* | T | 13 | 28 | 0.7 | 0.86 | 4.35 | 16.71 | 4.93 |
| Bees | *Andrena carantonica* | NL | 91 | 241 | 0.76 | 0.73 | 120.51 | 25.02 | -19.78 |
| Bees | *Andrena chrysosceles* | NL | 36 | 185 | 0.86 | 0.8 | 164.29 | 23.77 | -32.72 |
| Bees | *Andrena cineraria* | NL | 47 | 151 | 0.81 | 0.78 | 103.03 | 37.26 | 0.51 |
| Bees | *Andrena clarkella* | NL | 47 | 146 | 0.82 | 0.78 | 31.92 | 14.21 | 0.32 |
| Bees | *Andrena dorsata* | NL | 37 | 167 | 0.84 | 0.81 | 59.84 | -1.62 | -22.79 |
| Bees | *Andrena falsifica* | NL | 10 | 6 | 0.8 | 0.9 | -57.14 | 31.18 | 3.58 |
| Bees | *Andrena flavipes* | NL | 74 | 381 | 0.8 | 0.73 | 124.53 | 20.06 | -32.63 |
| Bees | *Andrena florea* | NL | 23 | 81 | 0.96 | 0.92 | 255.56 | 33.73 | -91.54 |
| Bees | *Andrena fucata* | NL | 43 | 80 | 0.75 | 0.78 | 230.36 | 56.21 | -2.69 |
| Bees | *Andrena fulva* | NL | 81 | 268 | 0.78 | 0.86 | 285.19 | 63.25 | 21.98 |
| Bees | *Andrena fulvago* | T | 12 | 17 | 0.95 | 0.95 | 28.21 | -3.28 | 5.17 |
| Bees | *Andrena fulvida* | T | 21 | 23 | 0.8 | 0.71 | 147.42 | 9.98 | -23.22 |
| Bees | *Andrena fuscipes* | VU | 120 | 132 | 0.81 | 0.87 | 32.67 | 31.03 | 5.49 |
| Bees | *Andrena gravida* | T | 36 | 57 | 0.83 | 0.87 | 83.56 | 21.45 | -13.2 |
| Bees | *Andrena haemorrhoa* | NL | 118 | 442 | 0.75 | 0.64 | 192.17 | 15.49 | -24.11 |
| Bees | *Andrena hattorfiana* | T | 26 | 22 | 0.93 | 0.94 | -14.29 | 9.36 | -0.33 |
| Bees | *Andrena helvola* | NL | 13 | 58 | 0.94 | 0.83 | 825 | 109.38 | -61.86 |
| Bees | *Andrena humilis* | VU | 39 | 40 | 0.88 | 0.89 | 45.78 | 31.99 | 8.56 |
| Bees | *Andrena labialis* | T | 26 | 49 | 0.92 | 0.88 | 159.57 | 3.29 | -70.65 |
| Bees | *Andrena labiata* | NL | 22 | 66 | 0.85 | 0.81 | 247.27 | 39.54 | -21.78 |
| Bees | *Andrena lapponica* | NL | 29 | 38 | 0.81 | 0.9 | 192.59 | -9.67 | -16.29 |
| Bees | *Andrena lathyri* | NL | 9 | 12 | 0.97 | 0.95 | 67.44 | 55.93 | 0.64 |
| Bees | *Andrena minutula* | NL | 42 | 117 | 0.87 | 0.82 | 52.56 | 47.24 | -23.98 |
| Bees | *Andrena minutuloides* | NL | 6 | 13 | 0.9 | 0.93 | 625 | 15.17 | 8.28 |
| Bees | *Andrena mitis* | NL | 19 | 46 | 0.88 | 0.89 | 247.22 | 71.7 | -35.84 |
| Bees | *Andrena nigriceps* | VU | 23 | 23 | 0.86 | 0.88 | -8.93 | -5.12 | -21.21 |
| Bees | *Andrena nigroaenea* | NL | 75 | 127 | 0.77 | 0.78 | -3.68 | 36.93 | -4.02 |
| Bees | *Andrena nitida* | NL | 77 | 219 | 0.82 | 0.72 | 31.03 | 15.15 | 18.47 |
| Bees | *Andrena ovatula* | VU | 56 | 71 | 0.86 | 0.85 | 6.67 | 26.26 | 9.86 |
| Bees | *Andrena pilipes* | T | 39 | 13 | 0.82 | 0.95 | 18.18 | 10.9 | 3.59 |
| Bees | *Andrena praecox* | NL | 64 | 157 | 0.73 | 0.75 | 45.58 | 15.72 | 7.36 |
| Bees | *Andrena proxima* | NL | 15 | 88 | 0.87 | 0.84 | 153.85 | 54.16 | -63.93 |
| Bees | *Andrena ruficrus* | NL | 40 | 35 | 0.84 | 0.88 | -16.91 | -51.44 | -23.01 |
| Bees | *Andrena semilaevis* | NL | 31 | 29 | 0.82 | 0.94 | -8.24 | 33.83 | 12.43 |
| Bees | *Andrena subopaca* | NL | 79 | 219 | 0.76 | 0.73 | 100 | 20.93 | -31.74 |
| Bees | *Andrena synadelpha* | NL | 7 | 40 | 0.98 | 0.85 | 566.67 | 131.52 | -2.65 |
| Bees | *Andrena tibialis* | VU | 55 | 95 | 0.81 | 0.78 | 31.77 | 40.14 | -12.35 |
| Bees | *Andrena vaga* | NL | 41 | 245 | 0.82 | 0.75 | 110 | 32.78 | 6.92 |
| Bees | *Andrena varians* | VU | 53 | 36 | 0.78 | 0.71 | -18.94 | 54.38 | -11.26 |
| Bees | *Andrena ventralis* | NL | 21 | 119 | 0.87 | 0.8 | 218.46 | 11.83 | -9.73 |
| Bees | *Andrena wilkella* | VU | 51 | 78 | 0.79 | 0.79 | 105.22 | 24.31 | -20.9 |
| Bees | *Anthidium manicatum* | NL | 31 | 171 | 0.89 | 0.77 | 256.45 | 45.25 | -35.71 |
| Bees | *Anthidium punctatum* | VU | 12 | 25 | 0.84 | 0.94 | 29.17 | 63.85 | -29.19 |
| Bees | *Anthidium strigatum* | NL | 22 | 81 | 0.9 | 0.88 | 262.16 | 10.5 | 13.84 |
| Bees | *Anthophora furcata* | VU | 23 | 46 | 0.89 | 0.85 | 152.08 | 43.13 | -60.22 |
| Bees | *Anthophora plumipes* | NL | 39 | 151 | 0.84 | 0.82 | 135.71 | 10.25 | -40.2 |
| Bees | *Anthophora quadrimaculata* | VU | 15 | 20 | 0.89 | 0.98 | 20 | -18.52 | 24.73 |
| Bees | *Anthophora retusa* | T | 22 | 12 | 0.83 | 0.75 | 94.74 | 18.7 | 16.22 |
| Bees | *Bombus bohemicus* | NL | 70 | 90 | 0.8 | 0.73 | 0 | -8.38 | -32.47 |
| Bees | *Bombus campestris* | NL | 90 | 196 | 0.78 | 0.77 | 58.59 | -1.56 | -55.38 |
| Bees | *Bombus cryptarum* | NL | 20 | 16 | 0.81 | 0.66 | 278.18 | 17.44 | 53.2 |
| Bees | *Bombus hortorum* | NL | 76 | 232 | 0.78 | 0.74 | 118.49 | 18.49 | -22.19 |
| Bees | *Bombus humilis* | T | 33 | 10 | 0.87 | 0.81 | -48.81 | 41.11 | 21.58 |
| Bees | *Bombus hypnorum* | NL | 46 | 295 | 0.81 | 0.74 | 67.83 | -45.09 | -50.32 |
| Bees | *Bombus jonellus* | VU | 35 | 93 | 0.77 | 0.82 | 198.33 | -63.88 | -26.07 |
| Bees | *Bombus lapidarius* | NL | 115 | 511 | 0.79 | 0.66 | 126.09 | -17.12 | -17.94 |
| Bees | *Bombus lucorum* | NL | 82 | 305 | 0.79 | 0.69 | 154.84 | -2.07 | -36.03 |
| Bees | *Bombus magnus* | T | 30 | 46 | 0.79 | 0.9 | 248.89 | -13.6 | -12.58 |
| Bees | *Bombus muscorum* | T | 66 | 38 | 0.73 | 0.92 | 20 | 28.85 | -6.29 |
| Bees | *Bombus norvegicus* | NL | 20 | 26 | 0.82 | 0.77 | 119.51 | 18.79 | -50.28 |
| Bees | *Bombus pascuorum* | NL | 165 | 657 | 0.7 | 0.59 | 125 | -3.83 | -21.34 |
| Bees | *Bombus pratorum* | NL | 89 | 436 | 0.76 | 0.66 | 157.72 | 30.67 | -44.32 |
| Bees | *Bombus ruderarius* | VU | 57 | 89 | 0.79 | 0.94 | -5.56 | -65.47 | -141.69 |
| Bees | *Bombus sylvestris* | NL | 62 | 170 | 0.75 | 0.74 | 111.11 | 21.88 | -33.21 |
| Bees | *Bombus terrestris* | NL | 97 | 490 | 0.75 | 0.65 | 140.67 | 1.33 | -2.67 |
| Bees | *Bombus vestalis* | NL | 36 | 115 | 0.88 | 0.75 | 220 | 56.94 | -0.15 |
| Bees | *Bombus veteranus* | T | 44 | 6 | 0.77 | 0.95 | -63.03 | -83.93 | -113.42 |
| Bees | *Ceratina cyanea* | NL | 12 | 18 | 0.86 | 0.94 | -34.69 | 5.66 | 13.82 |
| Bees | *Chelostoma campanularum* | VU | 36 | 53 | 0.9 | 0.87 | 110.61 | 35.24 | -13.65 |
| Bees | *Chelostoma distinctum* | VU | 19 | 6 | 0.97 | 0.97 | -35.71 | 8.73 | 2.86 |
| Bees | *Chelostoma florisomne* | VU | 50 | 76 | 0.86 | 0.81 | 91.3 | 30.42 | -20.99 |
| Bees | *Chelostoma rapunculi* | NL | 55 | 106 | 0.86 | 0.81 | 96.7 | 43.16 | -4.47 |
| Bees | *Coelioxys elongata* | T | 10 | 11 | 0.78 | 0.68 | -25.27 | -26.52 | -101.5 |
| Bees | *Coelioxys inermis* | T | 30 | 52 | 0.8 | 0.83 | 126.09 | 20.8 | -72.77 |
| Bees | *Coelioxys mandibularis* | VU | 29 | 46 | 0.86 | 0.94 | 83.33 | -8.72 | -32.87 |
| Bees | *Coelioxys quadridentata* | T | 23 | 11 | 0.81 | 0.9 | -29.49 | 133.5 | 41.85 |
| Bees | *Colletes cunicularius* | NL | 42 | 188 | 0.79 | 0.74 | 234.85 | -24.22 | 42.01 |
| Bees | *Colletes daviesanus* | NL | 72 | 193 | 0.8 | 0.74 | 61.15 | 48.77 | -9.06 |
| Bees | *Colletes fodiens* | NL | 31 | 142 | 0.84 | 0.8 | 190.2 | -9.36 | -30.68 |
| Bees | *Colletes halophilus* | NL | 7 | 57 | 0.97 | 0.95 | 125.93 | -10.11 | 14.76 |
| Bees | *Colletes impunctatus* | SE | 5 | 5 | 0.91 | 0.9 | 242.86 | -44.67 | -57.2 |
| Bees | *Colletes marginatus* | NL | 16 | 35 | 0.7 | 0.84 | 1.16 | 2.08 | 47.67 |
| Bees | *Colletes succinctus* | NL | 46 | 100 | 0.82 | 0.89 | 44.44 | 60.6 | 27.24 |
| Bees | *Dasypoda hirtipes* | NL | 89 | 277 | 0.79 | 0.76 | 87.01 | 30.22 | -2.86 |
| Bees | *Epeoloides coecutiens* | NL | 14 | 82 | 0.89 | 0.87 | -15.73 | 46.38 | 15.98 |
| Bees | *Epeolus cruciger* | NL | 44 | 129 | 0.78 | 0.86 | 182.09 | 18.57 | 8.7 |
| Bees | *Epeolus variegatus* | NL | 18 | 117 | 0.79 | 0.82 | 142.62 | 10.04 | -17.75 |
| Bees | *Eucera longicornis* | T | 25 | 8 | 0.94 | 0.76 | 89.13 | 6.07 | 11.3 |
| Bees | *Halictus confusus* | NL | 61 | 111 | 0.83 | 0.81 | 57.14 | 27.05 | 15.88 |
| Bees | *Halictus rubicundus* | NL | 102 | 204 | 0.75 | 0.76 | 107 | 56.38 | -10.11 |
| Bees | *Halictus tumulorum* | NL | 73 | 290 | 0.74 | 0.71 | 125 | 15.04 | -48.92 |
| Bees | *Heriades truncorum* | NL | 61 | 135 | 0.83 | 0.78 | 32.76 | 47.02 | -9.13 |
| Bees | *Hylaeus annularis* | NL | 44 | 58 | 0.82 | 0.85 | 11.97 | -26.29 | 13.43 |
| Bees | *Hylaeus brevicornis* | NL | 61 | 76 | 0.81 | 0.8 | 46.34 | -11.42 | 22.97 |
| Bees | *Hylaeus communis* | NL | 92 | 236 | 0.75 | 0.76 | 19.07 | 22.15 | -8.42 |
| Bees | *Hylaeus confusus* | NL | 65 | 166 | 0.81 | 0.78 | 52.25 | 34.28 | -0.36 |
| Bees | *Hylaeus gibbus* | NL | 63 | 83 | 0.75 | 0.78 | 125.96 | 30.25 | -5.04 |
| Bees | *Hylaeus hyalinatus* | NL | 64 | 142 | 0.8 | 0.81 | 73.5 | 2.53 | 1.19 |
| Bees | *Hylaeus pectoralis* | VU | 17 | 21 | 0.81 | 0.93 | -74.71 | -17.39 | -35.6 |
| Bees | *Hylaeus pictipes* | NL | 20 | 24 | 0.9 | 0.83 | 29.11 | 32.67 | 11.17 |
| Bees | *Hylaeus punctulatissimus* | NL | 10 | 17 | 0.88 | 0.88 | 91.58 | 6.18 | -6.65 |
| Bees | *Hylaeus rinki* | NL | 12 | 13 | 0.79 | 0.9 | -66.14 | 123.57 | 46.68 |
| Bees | *Hylaeus signatus* | NL | 22 | 60 | 0.86 | 0.83 | 172 | 14.36 | 21.01 |
| Bees | *Lasioglossum albipes* | NL | 91 | 91 | 0.69 | 0.79 | -31.22 | 66.44 | 28.12 |
| Bees | *Lasioglossum brevicorne* | VU | 19 | 18 | 0.8 | 0.89 | -41.94 | 51.78 | -3.66 |
| Bees | *Lasioglossum calceatum* | NL | 179 | 394 | 0.68 | 0.7 | 127.97 | 2.45 | -34.57 |
| Bees | *Lasioglossum fratellum* | NL | 8 | 18 | 0.98 | 0.91 | 88.89 | 32.21 | 24.14 |
| Bees | *Lasioglossum fulvicorne* | NL | 51 | 67 | 0.87 | 0.84 | 150.62 | 34.63 | -8.67 |
| Bees | *Lasioglossum laticeps* | NL | 14 | 32 | 0.94 | 0.88 | 571.43 | 70.8 | 10.54 |
| Bees | *Lasioglossum lativentre* | T | 25 | 12 | 0.87 | 0.96 | -8.93 | -26.98 | 8.66 |
| Bees | *Lasioglossum leucopus* | NL | 62 | 130 | 0.8 | 0.77 | 43.75 | 36.86 | 35.84 |
| Bees | *Lasioglossum leucozonium* | NL | 138 | 313 | 0.74 | 0.7 | 138.28 | 42.64 | -11.59 |
| Bees | *Lasioglossum lucidulum* | NL | 21 | 77 | 0.87 | 0.76 | 157.14 | 37.69 | -8.94 |
| Bees | *Lasioglossum malachurum* | VU | 22 | 41 | 0.95 | 0.89 | 90.32 | 30.11 | -18.77 |
| Bees | *Lasioglossum minutissimum* | NL | 24 | 80 | 0.86 | 0.74 | 271.8 | -27.91 | 28.3 |
| Bees | *Lasioglossum morio* | NL | 37 | 206 | 0.91 | 0.78 | 329.03 | 58.22 | -72.44 |
| Bees | *Lasioglossum nitidiusculum* | T | 30 | 8 | 0.89 | 0.78 | 55.81 | 68.3 | -46.56 |
| Bees | *Lasioglossum nitidulum* | NL | 20 | 33 | 0.91 | 0.89 | 86.05 | 38.6 | -13.12 |
| Bees | *Lasioglossum parvulum* | VU | 28 | 19 | 0.88 | 0.87 | -11.32 | -7.32 | 20.87 |
| Bees | *Lasioglossum pauxillum* | NL | 17 | 74 | 0.87 | 0.86 | 506.67 | 72.11 | -56.23 |
| Bees | *Lasioglossum prasinum* | NL | 49 | 28 | 0.78 | 0.9 | -30.82 | 20.35 | 8.61 |
| Bees | *Lasioglossum punctatissimum* | NL | 81 | 103 | 0.77 | 0.84 | 5.1 | 16.26 | -10.91 |
| Bees | *Lasioglossum quadrinotatulum* | NL | 47 | 46 | 0.86 | 0.84 | 18.56 | 35.64 | -14.46 |
| Bees | *Lasioglossum quadrinotatum* | T | 13 | 12 | 0.77 | 0.81 | 20.37 | 27.97 | 0.65 |
| Bees | *Lasioglossum rufitarse* | NL | 49 | 12 | 0.81 | 0.8 | -19.6 | -50.63 | -22.89 |
| Bees | *Lasioglossum sabulosum* | NL | 14 | 49 | 0.74 | 0.89 | 25.95 | 10.09 | 14.14 |
| Bees | *Lasioglossum semilucens* | NL | 27 | 56 | 0.88 | 0.78 | 71.83 | 24.5 | -47.26 |
| Bees | *Lasioglossum sexnotatum* | VU | 40 | 87 | 0.92 | 0.82 | 102.44 | 39.81 | -11.41 |
| Bees | *Lasioglossum sexstrigatum* | NL | 132 | 226 | 0.71 | 0.72 | 65.81 | 11.57 | -11.28 |
| Bees | *Lasioglossum tarsatum* | NL | 21 | 15 | 0.81 | 0.96 | 41.18 | 8.53 | -56.87 |
| Bees | *Lasioglossum villosulum* | NL | 103 | 176 | 0.76 | 0.76 | 60.19 | 29.07 | -17.19 |
| Bees | *Lasioglossum xanthopus* | VU | 17 | 26 | 0.92 | 0.93 | 75.68 | 108.49 | -43.36 |
| Bees | *Lasioglossum zonulum* | NL | 75 | 132 | 0.83 | 0.77 | 28.67 | 59.07 | -12.59 |
| Bees | *Macropis europaea* | NL | 61 | 196 | 0.81 | 0.76 | -6.56 | 16.59 | 2.63 |
| Bees | *Megachile analis* | VU | 5 | 10 | 0.62 | 0.89 | 196.55 | -14.94 | -6.98 |
| Bees | *Megachile centuncularis* | VU | 74 | 173 | 0.77 | 0.81 | 35.66 | -10.9 | -66.84 |
| Bees | *Megachile circumcincta* | T | 48 | 27 | 0.73 | 0.85 | -16.28 | 14.38 | -24.09 |
| Bees | *Megachile ericetorum* | VU | 21 | 83 | 0.94 | 0.85 | 459.38 | -16.94 | -72.56 |
| Bees | *Megachile lapponica* | NL | 43 | 25 | 0.82 | 0.74 | -42.57 | 34.19 | -12.99 |
| Bees | *Megachile leachella* | VU | 39 | 57 | 0.9 | 0.92 | 56.92 | -7.13 | -34.54 |
| Bees | *Megachile ligniseca* | T | 16 | 46 | 0.82 | 0.88 | -8.49 | -8.57 | -24.1 |
| Bees | *Megachile maritima* | T | 29 | 20 | 0.89 | 0.99 | -52.38 | 40.93 | -5.4 |
| Bees | *Megachile versicolor* | NL | 34 | 105 | 0.84 | 0.79 | 133.33 | 12.25 | -5.53 |
| Bees | *Megachile willughbiella* | NL | 49 | 226 | 0.73 | 0.72 | 221.25 | 21.45 | 2.52 |
| Bees | *Melecta albifrons* | T | 22 | 38 | 0.86 | 0.8 | 173.53 | 86.87 | -16.65 |
| Bees | *Melitta haemorrhoidalis* | NL | 32 | 63 | 0.85 | 0.86 | 240 | 91.77 | 1.34 |
| Bees | *Melitta leporina* | VU | 21 | 67 | 0.77 | 0.89 | 262.5 | 6.52 | -120.04 |
| Bees | *Melitta nigricans* | NL | 15 | 72 | 0.81 | 0.84 | 92.31 | 43.13 | -14.69 |
| Bees | *Melitta tricincta* | VU | 12 | 19 | 0.96 | 0.86 | 4.55 | 32.97 | -57.4 |
| Bees | *Nomada alboguttata* | NL | 69 | 167 | 0.8 | 0.77 | 14.71 | 14.08 | -11.48 |
| Bees | *Nomada bifasciata* | VU | 27 | 31 | 0.91 | 0.91 | -28.41 | 36.74 | 0.11 |
| Bees | *Nomada fabriciana* | NL | 44 | 148 | 0.84 | 0.82 | 60.61 | 24.04 | -30.5 |
| Bees | *Nomada ferruginata* | VU | 20 | 88 | 0.83 | 0.8 | 155.26 | -3.87 | -20.89 |
| Bees | *Nomada flava* | NL | 115 | 272 | 0.79 | 0.7 | 161.61 | 18.92 | -19.73 |
| Bees | *Nomada flavoguttata* | NL | 49 | 156 | 0.82 | 0.78 | 37.39 | 14.32 | -36.42 |
| Bees | *Nomada flavopicta* | VU | 31 | 67 | 0.87 | 0.83 | 135.39 | 17.65 | -75.25 |
| Bees | *Nomada fucata* | NL | 36 | 160 | 0.88 | 0.82 | 125.3 | 28.4 | -34.43 |
| Bees | *Nomada fulvicornis* | T | 45 | 48 | 0.87 | 0.77 | 27.42 | 80.85 | -53.1 |
| Bees | *Nomada fuscicornis* | T | 24 | 23 | 0.9 | 0.91 | 17.31 | 70.9 | -18.02 |
| Bees | *Nomada goodeniana* | VU | 52 | 140 | 0.81 | 0.78 | 34.33 | 52.39 | 8.13 |
| Bees | *Nomada integra* | T | 23 | 10 | 0.79 | 0.68 | 51.35 | 5.94 | 7.33 |
| Bees | *Nomada lathburiana* | VU | 47 | 181 | 0.8 | 0.81 | 61.72 | 30.39 | 3.23 |
| Bees | *Nomada leucophthalma* | VU | 25 | 72 | 0.78 | 0.82 | 300 | 46.25 | 0.99 |
| Bees | *Nomada marshamella* | NL | 62 | 159 | 0.74 | 0.76 | 81.58 | 34.23 | -38.58 |
| Bees | *Nomada obscura* | VU | 5 | 6 | 0.84 | 0.96 | 150 | -96.62 | -9.15 |
| Bees | *Nomada panzeri* | NL | 59 | 124 | 0.77 | 0.78 | 0 | 12.21 | -30.16 |
| Bees | *Nomada ruficornis* | NL | 85 | 248 | 0.8 | 0.75 | 176.09 | 32.59 | -3.85 |
| Bees | *Nomada rufipes* | NL | 137 | 138 | 0.79 | 0.86 | 14.89 | 44.08 | 10.16 |
| Bees | *Nomada sheppardana* | NL | 66 | 154 | 0.82 | 0.79 | 92.86 | 16.71 | 12.5 |
| Bees | *Nomada signata* | NL | 49 | 98 | 0.86 | 0.79 | 197.59 | 5.16 | -31.28 |
| Bees | *Nomada similis* | VU | 15 | 19 | 0.91 | 0.9 | -5.26 | 52.88 | -5.63 |
| Bees | *Nomada striata* | T | 39 | 38 | 0.83 | 0.83 | 54 | 19.44 | 8.53 |
| Bees | *Nomada succincta* | NL | 76 | 145 | 0.81 | 0.8 | -9.14 | 26.01 | -11.39 |
| Bees | *Osmia aurulenta* | VU | 10 | 16 | 0.9 | 0.96 | 15.56 | -73.35 | 0.74 |
| Bees | *Osmia caerulescens* | VU | 52 | 65 | 0.82 | 0.8 | 106.93 | 17.15 | -6.51 |
| Bees | *Osmia claviventris* | NL | 28 | 40 | 0.86 | 0.85 | 90 | 28.33 | 6.6 |
| Bees | *Osmia cornuta* | VU | 27 | 66 | 0.9 | 0.78 | 100 | -0.11 | -40.11 |
| Bees | *Osmia leaiana* | T | 19 | 15 | 0.89 | 0.67 | 168.75 | 24.07 | 22.1 |
| Bees | *Osmia leucomelana* | NL | 28 | 45 | 0.89 | 0.79 | 90.91 | 27.53 | -8.42 |
| Bees | *Osmia niveata* | T | 36 | 28 | 0.92 | 0.85 | 51.28 | 1.2 | 1.3 |
| Bees | *Osmia rufa* | NL | 81 | 337 | 0.78 | 0.68 | 152.54 | 23.72 | -22.03 |
| Bees | *Osmia uncinata* | NL | 6 | 34 | 0.82 | 0.9 | 281.82 | 69.42 | 9.07 |
| Bees | *Panurgus banksianus* | NL | 64 | 72 | 0.83 | 0.82 | 1.17 | 47.42 | 8.91 |
| Bees | *Panurgus calcaratus* | NL | 68 | 147 | 0.8 | 0.83 | 73.04 | 40.89 | 25.2 |
| Bees | *Sphecodes albilabris* | NL | 21 | 174 | 0.92 | 0.8 | 294 | -37.36 | 61.02 |
| Bees | *Sphecodes crassus* | NL | 37 | 114 | 0.78 | 0.74 | 55.91 | -39.61 | -52.29 |
| Bees | *Sphecodes ephippius* | VU | 53 | 120 | 0.78 | 0.81 | 9.09 | -3.62 | 5.95 |
| Bees | *Sphecodes ferruginatus* | VU | 12 | 11 | 0.98 | 0.9 | 172 | 20.06 | 3.29 |
| Bees | *Sphecodes geoffrellus* | NL | 41 | 92 | 0.81 | 0.76 | 61.39 | -7.36 | -24.52 |
| Bees | *Sphecodes gibbus* | NL | 46 | 107 | 0.85 | 0.79 | 106.32 | 58.27 | 21.75 |
| Bees | *Sphecodes hyalinatus* | NL | 12 | 7 | 0.98 | 0.97 | 26.32 | 27.3 | 9.19 |
| Bees | *Sphecodes longulus* | NL | 29 | 94 | 0.77 | 0.78 | 408.57 | 31 | -14.04 |
| Bees | *Sphecodes marginatus* | NL | 25 | 52 | 0.75 | 0.79 | 404.76 | -29.33 | -21.75 |
| Bees | *Sphecodes miniatus* | NL | 39 | 142 | 0.8 | 0.76 | 47.13 | 10.04 | -40.05 |
| Bees | *Sphecodes monilicornis* | NL | 95 | 234 | 0.72 | 0.75 | 40 | 18.57 | -34.36 |
| Bees | *Sphecodes pellucidus* | NL | 63 | 184 | 0.77 | 0.77 | 107.08 | 9.13 | -7.93 |
| Bees | *Sphecodes puncticeps* | NL | 33 | 67 | 0.82 | 0.78 | 42.73 | 7.66 | -46.92 |
| Bees | *Sphecodes reticulatus* | NL | 25 | 97 | 0.85 | 0.78 | 157.35 | 54.59 | -27.12 |
| Bees | *Sphecodes rubicundus* | T | 7 | 30 | 0.94 | 0.85 | 227.27 | 66.11 | -12.26 |
| Bees | *Stelis breviuscula* | VU | 25 | 27 | 0.9 | 0.79 | 25 | 40.55 | -7.52 |
| Bees | *Stelis ornatula* | VU | 19 | 11 | 0.82 | 0.83 | 115.49 | -0.85 | 25.45 |
| Butterflies | *Aglais urticae* | NL | 237 | 1518 | 0.67 | 0.5 | 213.22 | 44.71 | 38.15 |
| Butterflies | *Anthocharis cardamines* | NL | 174 | 1226 | 0.73 | 0.58 | 422.22 | 39.31 | 12.78 |
| Butterflies | *Apatura iris* | CR | 19 | 58 | 0.9 | 0.88 | 59.74 | 40.07 | 17.72 |
| Butterflies | *Aphantopus hyperantus* | NL | 182 | 845 | 0.74 | 0.68 | 231.8 | 21.37 | 29.65 |
| Butterflies | *Araschnia levana* | NL | 240 | 1340 | 0.76 | 0.55 | 230.32 | 57.51 | 2.85 |
| Butterflies | *Argynnis aglaja* | CR | 51 | 44 | 0.8 | 0.91 | 71.13 | 9.56 | 23.87 |
| Butterflies | *Argynnis niobe* | EN | 54 | 53 | 0.86 | 0.97 | -28.09 | 24.4 | -2.98 |
| Butterflies | *Argynnis paphia* | EX | 46 | 95 | 0.87 | 0.77 | 265.71 | 47.05 | 0.77 |
| Butterflies | *Boloria aquilonaris* | CR | 9 | 10 | 0.98 | 0.98 | 4.76 | -6.75 | 0.11 |
| Butterflies | *Boloria selene* | EN | 125 | 61 | 0.74 | 0.88 | 11.94 | 32.61 | -0.42 |
| Butterflies | *Callophrys rubi* | NL | 154 | 421 | 0.81 | 0.81 | 122.77 | 3.74 | 0.47 |
| Butterflies | *Carterocephalus palaemon* | VU | 46 | 116 | 0.88 | 0.94 | 11.69 | 9.73 | -1.7 |
| Butterflies | *Celastrina argiolus* | NL | 239 | 1418 | 0.71 | 0.53 | 241.36 | 40.77 | 0.23 |
| Butterflies | *Coenonympha pamphilus* | NL | 319 | 1058 | 0.63 | 0.61 | 141 | 57.59 | 30.26 |
| Butterflies | *Coenonympha tullia* | CR | 66 | 12 | 0.88 | 0.98 | -81.58 | 56.88 | 3.65 |
| Butterflies | *Colias croceus* | NA | 268 | 864 | 0.71 | 0.62 | 57.88 | -16.59 | -1.88 |
| Butterflies | *Colias hyale* | NA | 188 | 401 | 0.75 | 0.66 | 74.31 | 17.1 | 20.13 |
| Butterflies | *Cupido minimus* | EX | 5 | 5 | 0.98 | 0.88 | -60 | 20.98 | 0.41 |
| Butterflies | *Erynnis tages* | CR | 26 | 8 | 0.92 | 0.95 | 118.75 | -46.54 | -51.31 |
| Butterflies | *Gonepteryx rhamni* | NL | 238 | 1389 | 0.7 | 0.54 | 140.85 | 36.71 | 17.9 |
| Butterflies | *Hesperia comma* | EN | 106 | 116 | 0.78 | 0.91 | 23.13 | 58.13 | 23.71 |
| Butterflies | *Heteropterus morpheus* | EN | 10 | 26 | 0.88 | 0.97 | -69.03 | -35.53 | -3.38 |
| Butterflies | *Hipparchia semele* | SU | 195 | 347 | 0.71 | 0.83 | 30.8 | 9.87 | -9.59 |
| Butterflies | *Hipparchia statilinus* | CR | 8 | 8 | 0.98 | 0.99 | -86.96 | -18.15 | -2.07 |
| Butterflies | *Inachis io* | NL | 206 | 1552 | 0.68 | 0.51 | 125.23 | 12.28 | 16.13 |
| Butterflies | *Issoria lathonia* | VU | 195 | 299 | 0.7 | 0.78 | 43.89 | -5.89 | -3.65 |
| Butterflies | *Lasiommata megera* | NL | 250 | 1346 | 0.67 | 0.55 | 364.07 | 58.4 | 28.17 |
| Butterflies | *Leptidea sinapis* | SU | 17 | 30 | 0.81 | 0.89 | -5.46 | -1.75 | -1.29 |
| Butterflies | *Limenitis camilla* | EN | 83 | 109 | 0.81 | 0.89 | -22.42 | 18.13 | 24.32 |
| Butterflies | *Lycaena dispar* | CR | 24 | 18 | 0.98 | 0.93 | -35.42 | -9.41 | 1.76 |
| Butterflies | *Lycaena phlaeas* | NL | 309 | 1380 | 0.66 | 0.53 | 131.82 | 1.64 | 14.13 |
| Butterflies | *Lycaena tityrus* | VU | 166 | 263 | 0.78 | 0.87 | 40.39 | 29.68 | 12.23 |
| Butterflies | *Maculinea alcon* | EN | 93 | 100 | 0.82 | 0.92 | -16.13 | 1.67 | -0.74 |
| Butterflies | *Maniola jurtina* | NL | 296 | 1356 | 0.66 | 0.56 | 228.22 | 16.13 | 34.73 |
| Butterflies | *Melanargia galathea* | NA | 14 | 11 | 0.85 | 0.87 | 8.16 | -29.8 | 9.75 |
| Butterflies | *Melitaea athalia* | CR | 49 | 24 | 0.85 | 0.98 | -70.27 | 11.56 | -15 |
| Butterflies | *Neozephyrus quercus* | NL | 128 | 686 | 0.76 | 0.72 | 301.26 | -6.78 | 6.66 |
| Butterflies | *Nymphalis antiopa* | EX | 61 | 374 | 0.83 | 0.72 | 161.49 | 50.77 | -26.14 |
| Butterflies | *Nymphalis polychloros* | CR | 64 | 43 | 0.76 | 0.78 | -38.13 | 8.03 | -7.68 |
| Butterflies | *Ochlodes faunus* | SU | 224 | 927 | 0.7 | 0.65 | 184.02 | 12.21 | 15.6 |
| Butterflies | *Papilio machaon* | NL | 129 | 882 | 0.77 | 0.66 | 350.71 | 6.9 | 9.01 |
| Butterflies | *Pararge aegeria* | NL | 183 | 1433 | 0.72 | 0.52 | 361.41 | 31.82 | 18.28 |
| Butterflies | *Pieris brassicae* | NL | 223 | 1524 | 0.69 | 0.51 | 224.35 | 28.66 | 5.31 |
| Butterflies | *Pieris napi* | NL | 249 | 1542 | 0.69 | 0.51 | 107.79 | 18.33 | 27.21 |
| Butterflies | *Pieris rapae* | NL | 255 | 1555 | 0.67 | 0.5 | 144.98 | 29.86 | 33.74 |
| Butterflies | *Plebeius agestis* | SU | 121 | 532 | 0.8 | 0.74 | 120.83 | -6.65 | 19.49 |
| Butterflies | *Plebeius argus* | SU | 164 | 333 | 0.78 | 0.85 | 44.24 | 13.25 | 0.57 |
| Butterflies | *Plebeius optilete* | CR | 7 | 5 | 0.93 | 0.96 | -75 | -0.71 | 9.64 |
| Butterflies | *Polygonia c-album* | NL | 166 | 1444 | 0.8 | 0.53 | 160.91 | 32.75 | -9.03 |
| Butterflies | *Polyommatus coridon* | NA | 28 | 6 | 0.95 | 0.92 | -15.39 | -12.03 | 15.51 |
| Butterflies | *Polyommatus icarus* | NL | 261 | 1359 | 0.71 | 0.53 | 145.02 | 7.19 | 3 |
| Butterflies | *Polyommatus semiargus* | EX | 19 | 10 | 0.94 | 0.96 | -62.07 | -39.26 | -10.74 |
| Butterflies | *Pontia daplidice* | NA | 36 | 48 | 0.78 | 0.7 | 92.06 | 30.22 | 87.25 |
| Butterflies | *Pyrgus malvae* | EN | 86 | 64 | 0.76 | 0.87 | 12.34 | 17.46 | 17.17 |
| Butterflies | *Pyronia tithonus* | NL | 177 | 823 | 0.77 | 0.7 | 150 | 26.96 | 20.62 |
| Butterflies | *Satyrium ilicis* | EN | 114 | 108 | 0.8 | 0.9 | 8.43 | 12.91 | -1.51 |
| Butterflies | *Thecla betulae* | EN | 41 | 77 | 0.87 | 0.94 | 144.44 | 35.3 | -0.8 |
| Butterflies | *Thymelicus lineola* | NL | 179 | 1316 | 0.74 | 0.55 | 236.32 | 40.8 | 70.44 |
| Butterflies | *Thymelicus sylvestris* | NL | 125 | 551 | 0.79 | 0.7 | 352.38 | 29.62 | 7.38 |
| Butterflies | *Vanessa atalanta* | NA | 342 | 1558 | 0.66 | 0.51 | 15.54 | 52.61 | 59.1 |
| Butterflies | *Vanessa cardui* | NA | 265 | 1539 | 0.66 | 0.5 | 37.5 | 39.34 | 23.11 |
| Hoverflies | *Anasimyia contracta* | SE | 20 | 43 | 0.87 | 0.73 | 27.03 | 23.96 | -25.13 |
| Hoverflies | *Anasimyia interpuncta* | NL | 45 | 175 | 0.82 | 0.76 | 65.94 | 35.87 | 17.22 |
| Hoverflies | *Anasimyia lineata* | NL | 148 | 312 | 0.74 | 0.72 | 85.07 | 9.98 | 13.97 |
| Hoverflies | *Anasimyia transfuga* | NL | 53 | 101 | 0.81 | 0.79 | 19.73 | 19.5 | 6.31 |
| Hoverflies | *Baccha elongata* | NL | 98 | 269 | 0.8 | 0.75 | 81.88 | 2.55 | -20.2 |
| Hoverflies | *Brachyopa insensilis* | NL | 5 | 10 | 0.95 | 0.87 | 335.71 | 121.21 | -28.55 |
| Hoverflies | *Brachyopa pilosa* | NL | 12 | 70 | 0.95 | 0.84 | 473.91 | 51.57 | -9.09 |
| Hoverflies | *Brachyopa scutellaris* | NL | 22 | 64 | 0.85 | 0.89 | 376.74 | 23.88 | 3.92 |
| Hoverflies | *Brachypalpoides lentus* | NL | 25 | 134 | 0.88 | 0.81 | 300 | 45.83 | -8.23 |
| Hoverflies | *Brachypalpus laphriformis* | SE | 7 | 54 | 0.92 | 0.89 | 573.91 | 109.28 | -1.89 |
| Hoverflies | *Ceriana conopsoides* | NL | 11 | 49 | 0.76 | 0.87 | 124.74 | 63.72 | -20.03 |
| Hoverflies | *Chalcosyrphus nemorum* | NL | 32 | 223 | 0.9 | 0.79 | 224.64 | 52.67 | -16.16 |
| Hoverflies | *Chamaesyrphus lusitanicus* | NL | 8 | 8 | 0.97 | 0.87 | 134.78 | -93.76 | 51.72 |
| Hoverflies | *Cheilosia albipila* | NL | 30 | 189 | 0.86 | 0.76 | 215.71 | 42.72 | -14.35 |
| Hoverflies | *Cheilosia barbata* | NL | 13 | 7 | 0.95 | 0.96 | 37.5 | 5.88 | 8.78 |
| Hoverflies | *Cheilosia bergenstammi* | NL | 37 | 183 | 0.86 | 0.8 | 386.96 | -20.02 | 24.59 |
| Hoverflies | *Cheilosia canicularis* | NL | 6 | 24 | 0.96 | 0.96 | 412.5 | 35.98 | 7.79 |
| Hoverflies | *Cheilosia carbonaria* | NL | 10 | 36 | 0.88 | 0.85 | 17.86 | 29.93 | -27.01 |
| Hoverflies | *Cheilosia chrysocoma* | SE | 14 | 39 | 0.9 | 0.85 | -26.39 | 85.68 | 14.36 |
| Hoverflies | *Cheilosia cynocephala* | NL | 11 | 56 | 0.92 | 0.78 | 75 | 36.38 | -12.35 |
| Hoverflies | *Cheilosia fraterna* | NL | 46 | 111 | 0.77 | 0.77 | 73.68 | 22.37 | 16.64 |
| Hoverflies | *Cheilosia grossa* | NL | 24 | 85 | 0.89 | 0.81 | 287.69 | 0.86 | 33.46 |
| Hoverflies | *Cheilosia illustrata* | NL | 19 | 324 | 0.88 | 0.78 | 978.95 | 13.32 | -47.33 |
| Hoverflies | *Cheilosia impressa* | NL | 61 | 208 | 0.82 | 0.76 | 132.74 | 37.13 | -18.2 |
| Hoverflies | *Cheilosia latifrons* | NL | 32 | 44 | 0.74 | 0.8 | 61.18 | 0.33 | -7.57 |
| Hoverflies | *Cheilosia lenis* | NL | 6 | 9 | 0.92 | 0.99 | 225 | 4.42 | 2.31 |
| Hoverflies | *Cheilosia longula* | NL | 26 | 19 | 0.87 | 0.89 | -47.87 | -23.92 | 20.8 |
| Hoverflies | *Cheilosia mutabilis* | NL | 28 | 27 | 0.82 | 0.8 | 6.25 | 25.88 | -10.37 |
| Hoverflies | *Cheilosia pagana* | NL | 154 | 679 | 0.73 | 0.63 | 236.05 | 30.86 | 5.02 |
| Hoverflies | *Cheilosia proxima* | NL | 19 | 130 | 0.88 | 0.87 | 438.89 | 31.15 | -16.24 |
| Hoverflies | *Cheilosia ranunculi* | T | 147 | 586 | 0.75 | 0.63 | 175.57 | 26.96 | 1.18 |
| Hoverflies | *Cheilosia scutellata* | NL | 63 | 83 | 0.85 | 0.88 | 46.46 | -14.06 | -20.84 |
| Hoverflies | *Cheilosia semifasciata* | NL | 12 | 65 | 0.95 | 0.87 | 275.61 | 63.09 | 0.59 |
| Hoverflies | *Cheilosia urbana* | NL | 29 | 33 | 0.84 | 0.82 | -1.02 | 20.69 | 24.22 |
| Hoverflies | *Cheilosia uviformis* | NL | 19 | 14 | 0.91 | 0.77 | 159.18 | 81.84 | -5.35 |
| Hoverflies | *Cheilosia variabilis* | NL | 45 | 199 | 0.89 | 0.81 | 281.43 | 47.65 | -39.36 |
| Hoverflies | *Cheilosia velutina* | NL | 14 | 24 | 0.93 | 0.88 | 68.63 | 44.4 | 60.18 |
| Hoverflies | *Cheilosia vernalis* | NL | 94 | 209 | 0.74 | 0.75 | 88.74 | 9.25 | -10.32 |
| Hoverflies | *Chrysogaster cemiteriorum* | NL | 22 | 14 | 0.91 | 0.99 | -42.42 | -4.52 | 0.96 |
| Hoverflies | *Chrysogaster solstitialis* | NL | 29 | 152 | 0.83 | 0.83 | 497.73 | 63.51 | -11.85 |
| Hoverflies | *Chrysogaster virescens* | NL | 7 | 37 | 0.95 | 0.87 | 500 | 69.03 | 42.99 |
| Hoverflies | *Chrysotoxum arcuatum* | VU | 33 | 36 | 0.86 | 0.92 | 17.44 | 5.72 | -5.94 |
| Hoverflies | *Chrysotoxum bicinctum* | NL | 66 | 209 | 0.84 | 0.81 | 62.07 | 22.43 | 6.83 |
| Hoverflies | *Chrysotoxum cautum* | NL | 70 | 261 | 0.85 | 0.79 | 133.57 | 35.07 | 3.41 |
| Hoverflies | *Chrysotoxum festivum* | NL | 56 | 108 | 0.83 | 0.8 | 65.19 | 24.34 | 11.24 |
| Hoverflies | *Chrysotoxum octomaculatum* | VU | 19 | 6 | 0.91 | 0.98 | -79.17 | -10.44 | -0.69 |
| Hoverflies | *Chrysotoxum vernale* | NL | 55 | 63 | 0.86 | 0.88 | -14.1 | 25.91 | 6.01 |
| Hoverflies | *Chrysotoxum verralli* | NL | 6 | 22 | 0.96 | 0.88 | 232.43 | 47.7 | 10.63 |
| Hoverflies | *Criorhina asilica* | NL | 17 | 25 | 0.83 | 0.89 | 160.42 | 79.81 | 14.3 |
| Hoverflies | *Criorhina berberina* | NL | 46 | 200 | 0.86 | 0.79 | 187.32 | 34.17 | 1.12 |
| Hoverflies | *Criorhina floccosa* | NL | 6 | 51 | 0.9 | 0.84 | 1109.09 | 89.07 | -14.36 |
| Hoverflies | *Criorhina pachymera* | NL | 5 | 32 | 0.78 | 0.92 | 566.67 | 68.22 | -10.47 |
| Hoverflies | *Criorhina ranunculi* | NL | 5 | 19 | 0.88 | 0.91 | 500 | 35.42 | 12.5 |
| Hoverflies | *Dasysyrphus albostriatus* | NL | 112 | 344 | 0.82 | 0.74 | 250 | 21.1 | -6.58 |
| Hoverflies | *Dasysyrphus hilaris* | VU | 55 | 31 | 0.8 | 0.9 | -45.69 | 9.75 | 6.24 |
| Hoverflies | *Dasysyrphus pauxillus* | SE | 5 | 15 | 0.91 | 0.83 | 2225 | -25.74 | 99.07 |
| Hoverflies | *Dasysyrphus pinastri* | VU | 29 | 22 | 0.88 | 0.8 | -34.78 | -8.89 | 11.84 |
| Hoverflies | *Dasysyrphus tricinctus* | NL | 106 | 204 | 0.79 | 0.82 | 104.05 | 19.46 | -6.91 |
| Hoverflies | *Dasysyrphus venustus* | NL | 89 | 255 | 0.76 | 0.77 | 58.46 | 7.49 | 12.91 |
| Hoverflies | *Didea alneti* | SE | 11 | 34 | 0.89 | 0.81 | -5.1 | 8.55 | -8.16 |
| Hoverflies | *Didea fasciata* | NL | 51 | 173 | 0.82 | 0.84 | 135.92 | -12.61 | -28.02 |
| Hoverflies | *Didea intermedia* | NL | 35 | 89 | 0.86 | 0.82 | 132.05 | -5.51 | 5.49 |
| Hoverflies | *Epistrophe eligans* | NL | 80 | 370 | 0.81 | 0.73 | 150 | 8.87 | -20.52 |
| Hoverflies | *Epistrophe flava* | VU | 10 | 16 | 0.87 | 0.83 | 40.98 | -13.55 | -8.08 |
| Hoverflies | *Epistrophe grossulariae* | NL | 40 | 130 | 0.83 | 0.83 | 308.7 | 40.11 | -21.09 |
| Hoverflies | *Epistrophe nitidicollis* | NL | 72 | 243 | 0.8 | 0.75 | 40.1 | 11.15 | -4.49 |
| Hoverflies | *Episyrphus balteatus* | NL | 260 | 1173 | 0.65 | 0.54 | 152.09 | -17.74 | 16 |
| Hoverflies | *Eriozona syrphoides* | NL | 8 | 7 | 0.86 | 0.91 | -36.36 | 30.18 | 2.69 |
| Hoverflies | *Eristalinus aeneus* | NL | 30 | 99 | 0.95 | 0.9 | 107.32 | 34.94 | 28.57 |
| Hoverflies | *Eristalinus sepulchralis* | NL | 201 | 726 | 0.7 | 0.6 | 107.08 | 41.17 | 16.93 |
| Hoverflies | *Eristalis abusiva* | NL | 139 | 443 | 0.76 | 0.66 | 146.63 | 17.78 | 29.61 |
| Hoverflies | *Eristalis anthophorina* | NL | 28 | 20 | 0.89 | 0.92 | -58.28 | -9.21 | 11.54 |
| Hoverflies | *Eristalis arbustorum* | NL | 315 | 994 | 0.65 | 0.55 | 87.16 | -9.29 | 13.95 |
| Hoverflies | *Eristalis horticola* | NL | 142 | 687 | 0.78 | 0.65 | 202.78 | 1.66 | -13.87 |
| Hoverflies | *Eristalis intricaria* | NL | 248 | 655 | 0.68 | 0.63 | 148.52 | 16.76 | 21.16 |
| Hoverflies | *Eristalis nemorum* | NL | 208 | 872 | 0.71 | 0.58 | 400 | 23.62 | 14.46 |
| Hoverflies | *Eristalis pertinax* | NL | 199 | 1073 | 0.72 | 0.55 | 252.69 | -6.34 | -8.62 |
| Hoverflies | *Eristalis similis* | NL | 22 | 74 | 0.82 | 0.76 | 325 | 23.49 | 21.3 |
| Hoverflies | *Eristalis tenax* | NL | 217 | 1135 | 0.7 | 0.55 | 208.43 | 5.29 | 17.32 |
| Hoverflies | *Eumerus funeralis* | NL | 34 | 101 | 0.86 | 0.86 | 169.51 | -24.79 | 20.65 |
| Hoverflies | *Eumerus ornatus* | NL | 11 | 8 | 0.97 | 0.93 | 177.27 | 41.77 | 6.83 |
| Hoverflies | *Eumerus sogdianus* | VU | 15 | 18 | 0.94 | 0.86 | -41.73 | 17.02 | -8.36 |
| Hoverflies | *Eumerus strigatus* | NL | 71 | 185 | 0.7 | 0.79 | 51.35 | 82.94 | 55.63 |
| Hoverflies | *Eupeodes corollae* | NL | 254 | 816 | 0.67 | 0.59 | 86.43 | -0.44 | 14.49 |
| Hoverflies | *Eupeodes goeldlini* | NL | 12 | 24 | 0.81 | 0.84 | 117.31 | -85.64 | 27.39 |
| Hoverflies | *Eupeodes lapponicus* | NL | 21 | 79 | 0.83 | 0.84 | 489.47 | 50.46 | -25.64 |
| Hoverflies | *Eupeodes latifasciatus* | NL | 66 | 301 | 0.75 | 0.72 | 147.75 | 38.08 | -5.33 |
| Hoverflies | *Eupeodes luniger* | NL | 70 | 387 | 0.78 | 0.73 | 191.91 | -7.05 | -0.13 |
| Hoverflies | *Eupeodes nielseni* | NL | 5 | 21 | 0.76 | 0.86 | 239.39 | -9.61 | -37.49 |
| Hoverflies | *Ferdinandea cuprea* | NL | 37 | 175 | 0.9 | 0.76 | 247.62 | 25.31 | -29.74 |
| Hoverflies | *Helophilus hybridus* | NL | 80 | 370 | 0.76 | 0.67 | 115.85 | -14.26 | -4.4 |
| Hoverflies | *Helophilus pendulus* | NL | 321 | 1123 | 0.65 | 0.53 | 90.78 | 15.68 | 15.92 |
| Hoverflies | *Helophilus trivittatus* | NL | 202 | 959 | 0.69 | 0.56 | 144.19 | -2.51 | 19.54 |
| Hoverflies | *Heringia brevidens* | NL | 5 | 15 | 0.89 | 0.67 | 2937.5 | 87.85 | -41.81 |
| Hoverflies | *Heringia heringi* | NL | 17 | 36 | 0.76 | 0.82 | 164 | 65.97 | -34.34 |
| Hoverflies | *Heringia pubescens* | VU | 19 | 24 | 0.89 | 0.87 | 19.67 | -10.13 | -25.34 |
| Hoverflies | *Heringia vitripennis* | NL | 71 | 49 | 0.78 | 0.75 | -4.62 | 12.7 | -5.06 |
| Hoverflies | *Lejogaster metallina* | NL | 149 | 288 | 0.76 | 0.71 | 132.02 | 42.47 | 3.3 |
| Hoverflies | *Lejogaster tarsata* | NL | 39 | 75 | 0.85 | 0.85 | 94.95 | -17.37 | -2.23 |
| Hoverflies | *Lejops vittata* | NL | 11 | 7 | 0.84 | 0.99 | -92.05 | 88.47 | 58.59 |
| Hoverflies | *Leucozona laternaria* | SE | 31 | 16 | 0.82 | 0.86 | 252.83 | -4.79 | 18.11 |
| Hoverflies | *Leucozona lucorum* | NL | 33 | 80 | 0.91 | 0.82 | 157.9 | 40.11 | -5.48 |
| Hoverflies | *Megasyrphus erratica* | NL | 18 | 58 | 0.85 | 0.87 | 232.08 | -79.58 | -19.11 |
| Hoverflies | *Melangyna cincta* | NL | 52 | 207 | 0.83 | 0.79 | 105.51 | 11.72 | -5.69 |
| Hoverflies | *Melangyna lasiophthalma* | NL | 18 | 70 | 0.82 | 0.82 | 471.43 | 40.23 | -22.5 |
| Hoverflies | *Melangyna quadrimaculata* | NL | 12 | 18 | 0.89 | 0.95 | 28.89 | 44.94 | -13.39 |
| Hoverflies | *Melangyna umbellatarum* | NL | 41 | 121 | 0.86 | 0.81 | 113.95 | 28.16 | 7.25 |
| Hoverflies | *Melanogaster aerosa* | NL | 12 | 13 | 0.92 | 0.91 | 296.77 | -13.47 | 25.27 |
| Hoverflies | *Melanogaster hirtella* | NL | 136 | 454 | 0.73 | 0.68 | 170.55 | 11.84 | 22.97 |
| Hoverflies | *Melanogaster nuda* | NL | 43 | 97 | 0.85 | 0.77 | 93.1 | 9.97 | 8.3 |
| Hoverflies | *Melanostoma mellinum* | NL | 310 | 993 | 0.62 | 0.56 | 170.91 | -4.93 | 15.85 |
| Hoverflies | *Melanostoma scalare* | NL | 134 | 702 | 0.71 | 0.64 | 317.65 | 0.92 | 13.87 |
| Hoverflies | *Meligramma guttata* | NL | 29 | 54 | 0.92 | 0.87 | 104.92 | 42.07 | -8.93 |
| Hoverflies | *Meligramma triangulifera* | NL | 27 | 77 | 0.9 | 0.85 | 545.24 | 36.38 | -26.34 |
| Hoverflies | *Meliscaeva auricollis* | NL | 99 | 307 | 0.78 | 0.72 | 143.36 | -2.98 | -8.79 |
| Hoverflies | *Meliscaeva cinctella* | NL | 99 | 147 | 0.84 | 0.82 | 18.61 | 24.14 | 5.52 |
| Hoverflies | *Merodon equestris* | NL | 63 | 307 | 0.82 | 0.75 | 128.77 | 8 | 24.4 |
| Hoverflies | *Microdon analis* | NL | 12 | 52 | 0.81 | 0.87 | 161.29 | 30.5 | 2.95 |
| Hoverflies | *Microdon devius* | NL | 5 | 5 | 0.94 | 0.7 | 250 | -2.87 | -0.81 |
| Hoverflies | *Myathropa florea* | NL | 165 | 978 | 0.73 | 0.58 | 216.67 | 2.49 | -4.69 |
| Hoverflies | *Neoascia geniculata* | NL | 42 | 26 | 0.89 | 0.87 | 51.35 | 14.64 | 26.46 |
| Hoverflies | *Neoascia interrupta* | NL | 10 | 32 | 0.68 | 0.8 | 5.71 | 30.07 | -30.48 |
| Hoverflies | *Neoascia meticulosa* | NL | 32 | 113 | 0.82 | 0.77 | 136.07 | 70.75 | -1.26 |
| Hoverflies | *Neoascia obliqua* | NL | 5 | 45 | 0.93 | 0.87 | 1008.33 | -22.64 | 11.95 |
| Hoverflies | *Neoascia podagrica* | NL | 249 | 502 | 0.67 | 0.67 | 120.86 | 10.6 | 10.56 |
| Hoverflies | *Neoascia tenur* | NL | 72 | 269 | 0.78 | 0.74 | 72.02 | -8.28 | 6.23 |
| Hoverflies | *Orthonevra brevicornis* | NL | 10 | 26 | 0.91 | 0.81 | 127.5 | 47.93 | 16.3 |
| Hoverflies | *Orthonevra geniculata* | NL | 7 | 19 | 0.93 | 0.82 | -20 | -6.72 | -2 |
| Hoverflies | *Orthonevra intermedia* | NL | 8 | 40 | 0.69 | 0.83 | 329.63 | 117.71 | 27.55 |
| Hoverflies | *Orthonevra nobilis* | NL | 14 | 11 | 0.97 | 0.96 | 229.41 | 19.45 | 3.34 |
| Hoverflies | *Paragus haemorrhous* | NL | 34 | 215 | 0.75 | 0.8 | 387.27 | 38.12 | 14.22 |
| Hoverflies | *Paragus pecchiolii* | NL | 6 | 18 | 0.93 | 0.97 | 273.68 | 8.95 | -1.62 |
| Hoverflies | *Parasyrphus annulatus* | NL | 9 | 52 | 0.93 | 0.92 | 63.74 | -1.99 | 2.16 |
| Hoverflies | *Parasyrphus lineolus* | NL | 34 | 31 | 0.8 | 0.87 | 74.29 | -8.37 | 10.97 |
| Hoverflies | *Parasyrphus malinellus* | SE | 27 | 35 | 0.9 | 0.89 | 92.73 | 5.64 | -4.24 |
| Hoverflies | *Parasyrphus punctulatus* | NL | 81 | 160 | 0.86 | 0.84 | 2.37 | 10.32 | -1.15 |
| Hoverflies | *Parasyrphus vittiger* | VU | 60 | 10 | 0.84 | 0.88 | -9.52 | -8.5 | 29.66 |
| Hoverflies | *Parhelophilus consimilis* | NL | 10 | 11 | 0.81 | 0.87 | 1022.22 | -2.44 | 36.09 |
| Hoverflies | *Parhelophilus frutetorum* | NL | 28 | 134 | 0.87 | 0.77 | 240.82 | 82.14 | -10.96 |
| Hoverflies | *Parhelophilus versicolor* | NL | 67 | 225 | 0.82 | 0.75 | 95.75 | -4.85 | 9.73 |
| Hoverflies | *Pelecocera tricincta* | NL | 13 | 39 | 0.94 | 0.96 | -22.86 | -15.59 | 11.6 |
| Hoverflies | *Pipiza austriaca* | SE | 35 | 14 | 0.87 | 0.9 | -17.14 | -28.22 | 7.92 |
| Hoverflies | *Pipiza bimaculata* | NL | 34 | 121 | 0.83 | 0.8 | 91.84 | 73.09 | 20.52 |
| Hoverflies | *Pipiza fenestrata* | NL | 24 | 12 | 0.82 | 0.84 | 92.16 | 14.97 | 37.03 |
| Hoverflies | *Pipiza lugubris* | NL | 19 | 68 | 0.8 | 0.83 | 178.67 | 34.28 | -5.67 |
| Hoverflies | *Pipiza luteitarsis* | VU | 15 | 18 | 0.82 | 0.85 | 14.46 | 22.21 | -19.36 |
| Hoverflies | *Pipiza noctiluca* | NL | 57 | 176 | 0.81 | 0.82 | 64.57 | 23.08 | -23.89 |
| Hoverflies | *Pipiza quadrimaculata* | VU | 22 | 6 | 0.92 | 0.87 | -82.2 | -119.93 | -5.69 |
| Hoverflies | *Pipizella viduata* | NL | 95 | 272 | 0.78 | 0.77 | 63.25 | 8.81 | -27.25 |
| Hoverflies | *Pipizella virens* | NL | 8 | 7 | 0.97 | 0.97 | 77.78 | -3.99 | 6.84 |
| Hoverflies | *Platycheirus albimanus* | NL | 194 | 726 | 0.76 | 0.62 | 156.48 | 11.37 | 8.12 |
| Hoverflies | *Platycheirus ambiguus* | SE | 12 | 9 | 0.86 | 0.85 | 38.89 | 72.34 | -15.41 |
| Hoverflies | *Platycheirus angustatus* | NL | 117 | 319 | 0.8 | 0.68 | 64.87 | 47.24 | 34.74 |
| Hoverflies | *Platycheirus clypeatus* | NL | 244 | 670 | 0.7 | 0.61 | 84.85 | 16.94 | 19.35 |
| Hoverflies | *Platycheirus discimanus* | VU | 8 | 6 | 0.91 | 0.8 | -52.5 | -4.17 | -18.56 |
| Hoverflies | *Platycheirus fulviventris* | NL | 81 | 148 | 0.78 | 0.8 | 22.3 | -11.39 | 17.68 |
| Hoverflies | *Platycheirus immarginatus* | NL | 14 | 13 | 0.87 | 0.73 | -39.41 | -79.68 | -55.25 |
| Hoverflies | *Platycheirus manicatus* | NL | 68 | 60 | 0.76 | 0.84 | 81.25 | -19.87 | -22.63 |
| Hoverflies | *Platycheirus peltatus* | NL | 223 | 329 | 0.69 | 0.7 | 44.04 | 12.93 | 26.62 |
| Hoverflies | *Platycheirus scambus* | NL | 151 | 202 | 0.73 | 0.77 | 97.89 | 10.21 | 7.7 |
| Hoverflies | *Platycheirus scutatus* | NL | 172 | 412 | 0.75 | 0.67 | 207.09 | 24.87 | 11.54 |
| Hoverflies | *Pyrophaena granditarsa* | NL | 156 | 295 | 0.73 | 0.74 | 113.46 | 7.5 | -5.26 |
| Hoverflies | *Pyrophaena rosarum* | NL | 48 | 178 | 0.91 | 0.81 | 69.12 | 27.07 | -11.55 |
| Hoverflies | *Rhingia campestris* | NL | 235 | 814 | 0.69 | 0.6 | 235.63 | 19.75 | 44.97 |
| Hoverflies | *Riponnensia splendens* | NL | 5 | 12 | 0.9 | 0.94 | 2150 | 27.52 | 8.05 |
| Hoverflies | *Scaeva pyrastri* | NL | 177 | 505 | 0.71 | 0.66 | 122.17 | 5.82 | -13.44 |
| Hoverflies | *Scaeva selenitica* | NL | 69 | 416 | 0.77 | 0.69 | 245.08 | 15.37 | 0.29 |
| Hoverflies | *Sericomyia lappona* | NL | 6 | 13 | 0.81 | 0.88 | 1009.09 | 27.03 | 56.15 |
| Hoverflies | *Sericomyia silentis* | NL | 61 | 258 | 0.81 | 0.8 | 50.46 | -2.85 | -10.08 |
| Hoverflies | *Sphaerophoria batava* | NL | 37 | 149 | 0.81 | 0.86 | 163.27 | -45.8 | 3.48 |
| Hoverflies | *Sphaerophoria fatarum* | NL | 23 | 15 | 0.82 | 0.88 | 105.81 | -5 | 22.34 |
| Hoverflies | *Sphaerophoria interrupta* | NL | 21 | 37 | 0.83 | 0.94 | 165.39 | 6.85 | -18.99 |
| Hoverflies | *Sphaerophoria philanthus* | NL | 17 | 40 | 0.82 | 0.91 | -34.02 | -2.03 | 18.63 |
| Hoverflies | *Sphaerophoria rueppelli* | NL | 27 | 105 | 0.83 | 0.83 | 127.63 | 39.76 | 19.3 |
| Hoverflies | *Sphaerophoria scripta* | NL | 189 | 931 | 0.7 | 0.57 | 133.95 | -1.37 | 10.7 |
| Hoverflies | *Sphaerophoria taeniata* | NL | 26 | 46 | 0.82 | 0.78 | 159.21 | 48.54 | 43.82 |
| Hoverflies | *Sphaerophoria virgata* | SE | 6 | 18 | 0.85 | 0.95 | -41.76 | 62.9 | 0.23 |
| Hoverflies | *Sphegina clunipes* | NL | 30 | 61 | 0.92 | 0.85 | 494.6 | 44.16 | -26.37 |
| Hoverflies | *Sphegina elegans* | NL | 15 | 24 | 0.86 | 0.92 | 39.66 | 17.6 | 10.76 |
| Hoverflies | *Syritta pipiens* | NL | 277 | 1028 | 0.68 | 0.54 | 187.69 | -0.43 | 31.98 |
| Hoverflies | *Syrphus ribesii* | NL | 231 | 866 | 0.69 | 0.58 | 139.52 | -3.95 | -3.07 |
| Hoverflies | *Syrphus torvus* | NL | 163 | 449 | 0.74 | 0.69 | 171.6 | -8.33 | -9.23 |
| Hoverflies | *Syrphus vitripennis* | NL | 239 | 596 | 0.69 | 0.64 | 102.36 | -24.89 | -1.58 |
| Hoverflies | *Temnostoma bombylans* | NL | 13 | 130 | 0.93 | 0.81 | 411.63 | 84.36 | -33.19 |
| Hoverflies | *Temnostoma vespiforme* | NL | 14 | 78 | 0.87 | 0.86 | 348.39 | 71.79 | -26.21 |
| Hoverflies | *Trichopsomyia flavitarsis* | SE | 11 | 21 | 0.7 | 0.75 | 73.13 | 32.84 | -11.91 |
| Hoverflies | *Trichopsomyia lucida* | NL | 8 | 14 | 0.85 | 0.92 | 220 | 51.28 | -25.71 |
| Hoverflies | *Triglyphus primus* | SE | 17 | 24 | 0.85 | 0.9 | -21.77 | 43.3 | -25.9 |
| Hoverflies | *Tropidia scita* | NL | 122 | 464 | 0.73 | 0.67 | 112.5 | 7.76 | 18.27 |
| Hoverflies | *Volucella bombylans* | NL | 131 | 461 | 0.75 | 0.71 | 255.56 | 30.31 | -3.69 |
| Hoverflies | *Volucella pellucens* | NL | 109 | 514 | 0.78 | 0.7 | 186.67 | 0.79 | -17.43 |
| Hoverflies | *Volucella zonaria* | NL | 12 | 374 | 0.87 | 0.76 | 694.44 | 48.99 | -32.11 |
| Hoverflies | *Xanthandrus comtus* | NL | 33 | 120 | 0.73 | 0.74 | 75.26 | 10.28 | -13.65 |
| Hoverflies | *Xanthogramma citrofasciatum* | NL | 14 | 25 | 0.93 | 0.95 | 39.68 | 45.14 | -3.52 |
| Hoverflies | *Xanthogramma pedissequum* | NL | 39 | 258 | 0.89 | 0.8 | 198.29 | 23.89 | -17.84 |
| Hoverflies | *Xylota abiens* | SE | 24 | 24 | 0.91 | 0.81 | 98.88 | 14.17 | 4.85 |
| Hoverflies | *Xylota florum* | VU | 36 | 8 | 0.87 | 0.73 | 71.88 | -7.02 | 22.6 |
| Hoverflies | *Xylota meigeniana* | VU | 7 | 6 | 0.95 | 0.93 | -24 | -2.46 | -5.81 |
| Hoverflies | *Xylota segnis* | NL | 153 | 700 | 0.74 | 0.64 | 164.07 | 11.78 | -19.25 |
| Hoverflies | *Xylota sylvarum* | NL | 57 | 221 | 0.86 | 0.78 | 193.83 | 26.82 | -17.88 |
| Hoverflies | *Xylota tarda* | NL | 18 | 34 | 0.94 | 0.8 | 211.63 | 28.46 | -5.77 |
| Hoverflies | *Xylota xanthocnema* | NL | 10 | 31 | 0.93 | 0.91 | 240 | 57.8 | -5.85 |
| *Red list status – Bees and hoverflies: *T*, Threatened; *VU*, Vulnerable; *SE*, Sensitive; NL, Not considered threatened. – Butterflies: *EX*, Extinct (status in 2005); *CR,* Critically endangered; *EN,* endangered; *VU,* vulnerable; *SU,* Susceptible; *NL,* Not considered threatened; *NA,* Migrant or vagrant. | | | | | | | | | |

References

1. Peeters, T. M. & Reemer, M. *Bedreigde en verdwenen bijen in Nederland (Apidae sl) basisrapport met voorstel voor de rode lijst.* (2003).

2. Reemer, M., Renema, W., van Steenis, W., Zeegers, T. & Smit, J. in *De Nederlandse zweefvliegen Diptera: Syrphidae* 444 (National Natuurhistorisch Museum, Leiden, 2009).

| **Table S6. Generalized variance inflation factor (GVIF) analysis used to detect highly collinear terms and applied to all species traits included in the linear models. All terms show GVIF values below 3.2, which suggest no high colinearity between terms.** | | |
| --- | --- | --- |
| **Pollinator group** | **Predictor variable** | **GVIF** |
| *Bees* |  |  |
|  | Body size | 1.17 |
|  | Flight period | 1.30 |
|  | Habitat specialisation | 1.38 |
|  | Larval food preference | 1.13 |
|  | Range size P1 | 1.43 |
|  | Voltinism | 1.14 |
| *Butterflies* |  |  |
|  | Body size | 1.38 |
|  | Flight period | 1.85 |
|  | Habitat specialisation | 3.17 |
|  | Larval food dependence on nitrogen | 2.59 |
|  | Larval food preference | 1.15 |
|  | Range size P1 | 1.51 |
|  | Voltinism | 1.64 |
| *Hoverflies* |  |  |
|  | Body size | 1.10 |
|  | Flight period | 1.79 |
|  | Habitat specialisation | 1.24 |
|  | Larval food preference | 1.14 |
|  | Range size P1 | 1.78 |
|  | Voltinism | 1.64 |

**Figure S1.** In the left panel: The maps of distribution of collection effort over time in the Netherlands (resolution: 5 × 5 km grid cells). Light grey: grid cells where no species were collected. Coloured (red, blue or dark grey): grid cells where species had been sampled. The information is given for each of the two time periods analysed (TP1 and TP2) and for the three pollinator groups (bees, butterflies, hoverflies)*. The maps were created using the R “raster” package (https://cran.r-project.org/web/packages/raster/index.html). In the right panel the boxplots of latitudinal and longitudinal geographic distribution of the sampled grid cells is shown in comparison to the distribution of all grid cells in the study area for each pollinator group. The sampled grid cells cover similar latitudinal and longitudinal extents across the study area and time periods suggesting a similar coverage of environmental conditions.

*The bumblebee image in Figure S1 was created by the authors of this publication. The butterfly and hoverfly images for Figure S1 where modified to the grey scale from en.wikipedia.org/wiki/Coenonympha_tullia and en.wikipedia.org/wiki/Hoverfly respectively. These images are licensed under the Creative Commons Attribution-ShareAlike 4.0 International License. To view a copy of this license, visit http://creativecommons.org/licenses/by-sa/4.0/ or send a letter to Creative Commons, PO Box 1866, Mountain View, CA 94042, USA.
